# Plasma Metabolites Related to the Consumption of Different Types of Dairy Products and Their Association with New‐Onset Type 2 Diabetes: Analyses in the Fenland and EPIC‐Norfolk Studies, United Kingdom

**DOI:** 10.1002/mnfr.202300154

**Published:** 2023-12-06

**Authors:** Eirini Trichia, Albert Koulman, Isobel D. Stewart, Soren Brage, Simon J. Griffin, Julian L. Griffin, Kay‐Tee Khaw, Claudia Langenberg, Nicholas J. Wareham, Fumiaki Imamura, Nita G. Forouhi

**Affiliations:** ^1^ MRC Epidemiology Unit Institute of Metabolic Science University of Cambridge School of Clinical Medicine Cambridge CB2 0SL UK; ^2^ Department of Biochemistry University of Cambridge Cambridge CB2 1QW UK

**Keywords:** biomarkers, dairy, diabetes, metabolites, metabolomics

## Abstract

**Scope:**

To identify metabolites associated with habitual dairy consumption and investigate their associations with type 2 diabetes (T2D) risk.

**Methods and results:**

Metabolomics assays were conducted in the Fenland (*n* = 10,281) and EPIC‐Norfolk (*n* = 1,440) studies. Using 82 metabolites assessed in both studies, we developed metabolite scores to classify self‐reported consumption of milk, yogurt, cheese, butter, and total dairy (Fenland Study‐discovery set; *n* = 6035). Internal and external validity of the scores was evaluated (Fenland‐validation set, *n* = 4246; EPIC‐Norfolk, *n* = 1440). The study assessed associations between each metabolite score and T2D incidence in EPIC‐Norfolk (*n* = 641 cases; 16,350 person‐years). The scores classified low and high consumers for all dairy types with internal validity, and milk, butter, and total dairy with external validity. The scores were further associated with lower incident T2D: hazard ratios (95% confidence interval) per standard deviation: milk 0.71 (0.65, 0.77); butter 0.62 (0.57, 0.68); total dairy 0.66 (0.60, 0.72). These associations persisted after adjustment for known dairy‐fat biomarkers.

**Conclusion:**

Metabolite scores identified habitual consumers of milk, butter, and total dairy products, and were associated with lower T2D risk. These findings hold promise for identifying objective indicators of the physiological response to dairy consumption.

## Graphical abstract

Dairy foods may be relevant for type 2 diabetes prevention and assessed more objectively with blood markers. Blood biomarkers were in combination related to consumption of milk, butter, and total dairy products in the Fenland and EPIC‐Norfolk studies and are associated with lower type 2 diabetes risk. In Fenland Study, biomarkers are only related to yoghurt and cheese.

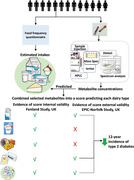

## 1 Introduction

Meta‐analyses of prospective cohort studies have reported diverse associations between the consumption of dairy products and type 2 diabetes (T2D): inverse associations for yogurt^[^ ^1^ ^]^ and butter,^[^ ^2^ ^]^ generally neutral associations for milk (total and full‐fat) and high‐fat dairy products and inconsistent, null or inverse associations for other dairy types, e.g., low‐fat milk, low‐fat dairy products, and cheese.^[^ ^1^ ^]^ The objective measurement and use of specific biomarkers of different types of dairy consumption could offer a complementary approach to subjective dietary assessment to help further elucidate the link between dairy products and health outcomes.

Biomarkers for the consumption of total and full‐fat dairy products have previously been identified to include odd‐chain saturated fatty acids (OCSFAs: pentadecanoate, C15:0; and heptadecanoate, C17:0), and *trans*‐palmitoleate (C16:1n7*t*).^[^ ^3^ ^]^ The use of metabolomics to identify metabolites associated with dairy consumption might further contribute to the objective assessment of the physiological response to dairy consumption. A few studies have aimed to identify novel candidate dairy biomarkers using metabolomics,^[^ ^4^, ^5^ ^]^ but limited evidence to date has precluded their establishment as dairy biomarkers. Issues include the lack of specificity of the identified biomarkers to individual dairy types,^[^ ^6^, ^7^ ^]^ limited generalizability to a general population because of inclusion of patient populations^[^ ^8^ ^]^ or those with high dairy consumption,^[^ ^7^ ^]^ and use of diverse biological samples and populations limiting comparability of results.^[^ ^5^, ^6^, ^7^, ^8^ ^]^

We hypothesized that a combination of selected metabolites could classify response to low and high consumption of different dairy types and be associated with T2D risk adding to the evidence from studies on self‐reported consumption and fatty acid biomarkers. We address these hypotheses by evaluating plasma metabolomics profiles in two independent, geographically‐similar, population‐based cohorts in the United Kingdom. We specifically aimed to develop and assess the validity of metabolite scores that could identify consumers of milk, yogurt, cheese, butter, and total dairy. We also evaluated utility of each metabolite score as a biomarker in comparison to the known fatty acid biomarkers.

## 2 Experimental Section

### 2.1 Study Design and Populations

**Figure 1:**
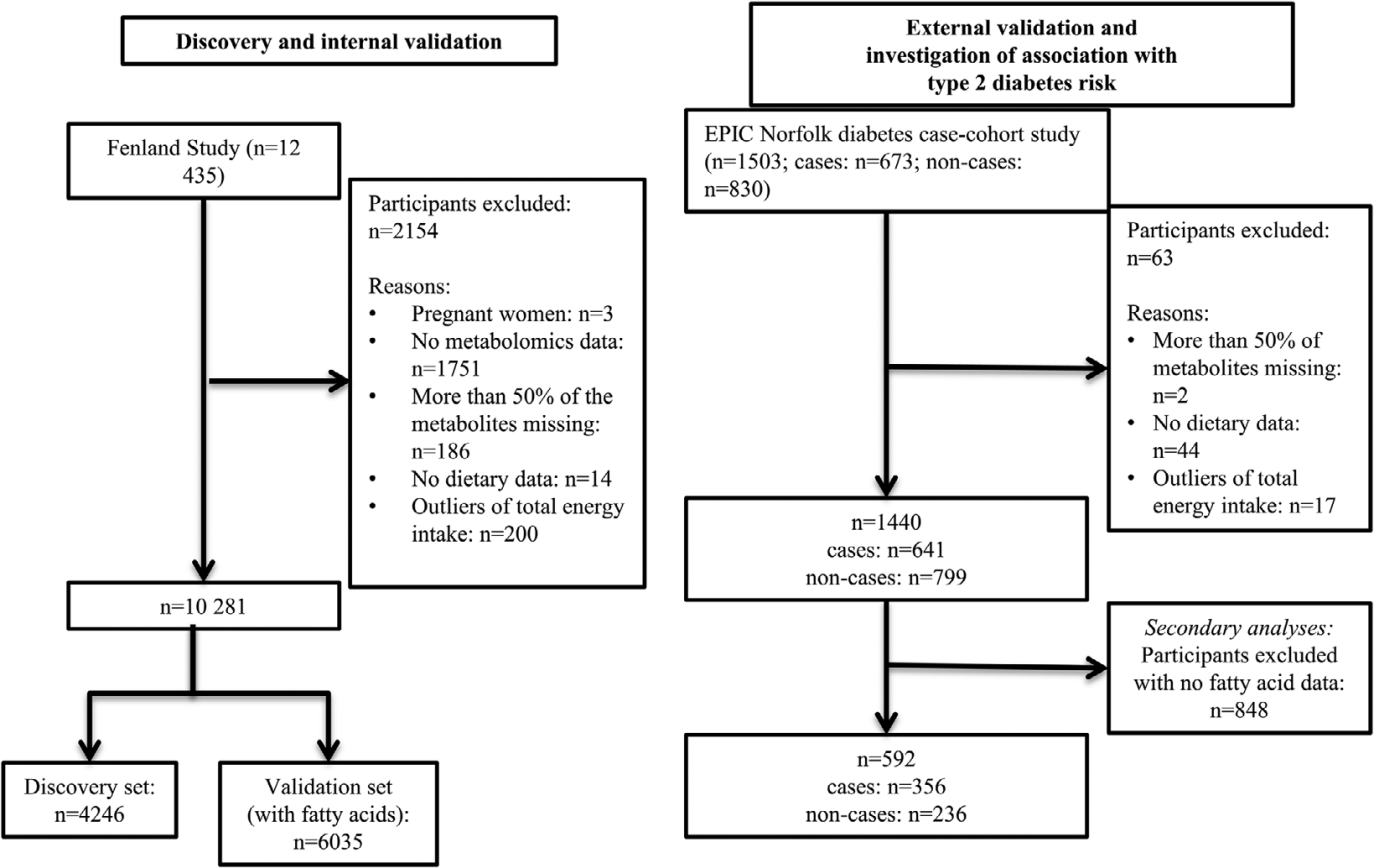
Flow diagram of the inclusion process of participants in the derivation, internal, and external validation sets (the Fenland Study and the EPIC Norfolk Study in the United Kingdom).

This study included two populations (**Figure** **1**): the Fenland Study and the European Prospective Investigation into Cancer and Nutrition, Norfolk (EPIC‐Norfolk) case‐cohort study. Briefly, the sudy evaluated participants in the Fenland Study to derive metabolite scores from metabolomics so that scores could classify between low and high consumption of each dairy type, to assess internal validity of each score, and to assess their utility additional to the dairy fat biomarkers. External validity of the metabolite scores was assessed similarly in EPIC‐Norfolk with and without the dairy fat biomarkers accounted for. In EPIC‐Norfolk the study further assessed associations of the metabolite scores with incident T2D.

The Fenland Study was a cohort study, which started in 2005 and recruited 12 435 adults born between 1950 and 1975. Recruitment via general practices and baseline assessment were carried out in Cambridge, Ely and Wisbech, Cambridgeshire, United Kingdom.^[^ ^9^, ^10^ ^]^ The study was approved by the Cambridge Local Ethics Committee. All participants provided written informed consent. The current sample included 10 281 participants after excluding participants with no metabolomics data (*n* = 1751) or more than 50% of the metabolites missing (*n* = 186), pregnant women (*n* = 3), or participants recording no dietary data (*n* = 14), or implausible energy intake (<800 or >4000 kcal day^−1^ among men; <500 or >3500 kcal day^−1^ among women; *n* = 200). The remaining participants were divided into a derivation set (*n* = 6035) and a validation set (*n* = 4246). The derivation set was selected as the set without data on plasma phospholipid fatty acids (details in the section below on fatty acid measurements). The validation set comprised a cohort subset randomly selected for plasma phospholipid fatty acid assays that provided data on OCSFAs (C15:0, C17:0), and C16:1n7*t*. Participants were evaluated in the validation set to assess the utility of the metabolite scores in addition to the fatty acids.

The EPIC‐Norfolk Study (DOI:10.22025/2019.10.105.00004) was a cohort study with baseline measurements between 1993 and 1997 among 25 639 adults recruited through general practices to attend clinical and dietary assessments in Norfolk, United Kingdom.^[^ ^11^ ^]^ All participants provided written informed consent, and the study was approved by the Norwich Local Ethics Committee (REC Ref: 98CN01). This current study evaluated data from a T2D case‐cohort study nested within the EPIC‐Norfolk Study, with 1503 participants (673 incident T2D cases) to examine biomarker–T2D associations. After excluding participants with no dietary data (*n* = 44), implausible energy intake (as described above; *n* = 17), or more than 50% of the metabolites missing (*n* = 2), the current analysis included 1440 participants (641 incident T2D cases). In the analysis involving fatty acids, participants with no fatty acid measurements (*n* = 848) were further excluded leaving 592 participants (356 incident T2D cases).

### 2.2 Metabolite and Fatty Acid Measurement

In the Fenland Study, targeted metabolomics profiling of fasting blood plasma samples was performed with a commercial kit (Absolute IDQ p180 kit, BIOCRATES Life Sciences AG, Innsbruck, Austria).^[^ ^12^, ^13^ ^]^ Lipids and acylcarnitines were measured using flow injection analysis mass spectrometry (MS; AB SCIEX 5500 Qtrap mass spectrometer, Sciex Ltd, Warrington, UK) in positive ionization mode and hexose in negative ionization mode. This method provided relative concentrations. Amino acids and biogenic amines were measured with UPLC‐MS (ultra‐performance liquid chromatography [Acquity UPLC, Waters ltd., Manchester, UK], coupled to the same spectrometer as above), for which internal standards and calibration lines were used to obtain absolute concentrations. A total of 187 metabolites were measured. After quality controls, 13 metabolites were excluded, leaving 174 metabolites: amino acids (*n* = 22), biogenic amines (*n* = 12), acylcarnitines (*n* = 40), phosphatidylcholines (PCs; *n* = 74), lysophosphatidylcholines (LPCs; *n* = 14), sphingomyelins (SMs; *n* = 11), and hexose.

In the EPIC‐Norfolk Study, untargeted metabolomics profiling was performed in non‐fasting plasma samples collected at baseline from 1503 participants in the T2D case‐cohort study nested within the EPIC Norfolk Study (DiscoveryHD4^®^ platform, Metabolon, Inc.).^[^ ^12^, ^13^ ^]^ After quality control procedures, 940 metabolites were assessed, of which 602 were annotated; relative concentrations were measured for all metabolite signals.

Of the two different metabolomics platforms used in the Fenland Study and the EPIC Norfolk Study, 82 metabolites present in both datasets (Supplemental Methods, Supporting Information) were identified and re‐annotated them in concordance with Biochemical Nomenclature^[^ ^14^ ^]^ (Supplemental Methods, Table S1, Supporting Information).

### 2.3 Fatty Acids Assays

Fatty acids of the plasma phospholipid fraction were measured in 4791 participants in the Fenland Study and in 592 participants in the EPIC‐Norfolk T2D case‐cohort study using GC as previously described (Supplementary Methods, Supporting Information).^[^ ^15^ ^]^ The fatty acid measurements were expressed as relative concentrations (mol%). Relative concentrations of C15:0, C17:0, and C16:1n7t from the Fenland Study and C15:0 and C17:0 from the EPIC‐Norfolk Study (C16:1n7t not available) were evaluated as covariates when developing metabolite scores from metabolites and also combined with metabolites to develop a score based on both metabolites and the fatty acids.

### 2.4 Dietary Assessment

In both studies, diet was assessed with a 130‐item semi‐quantitative food frequency questionnaire to estimate habitual dietary intakes over the past year. Participants were asked to choose one of the nine frequencies of dairy consumption ranging from “never or less than once/month” to “6 times per day” and provide more details on the type and amount of milk consumed. Questionnaire data were processed using in‐house software.^[^ ^16^ ^]^ The questionnaire validity was assessed against 7‐day food diaries in the EPIC‐Norfolk Study.^[^ ^17^ ^]^ Correlations between estimates from the questionnaires and the 7‐day food diaries were 0.56 for milk, 0.57 for yogurt, 0.33 for cheese, and 0.54 for butter.

### 2.5 Diabetes Case Ascertainment

Cases of incident T2D in the EPIC‐Norfolk Study occurring until July 31, 2006 were ascertained using a combination of information from self‐report and independent health records. Within the cohort, information was collected prospectively on self‐report of physician diagnosed diabetes or use of diabetes drugs. Data were also collected from record linkage with general practices or local hospital records, hospital admissions, and mortality data from the UK Office of National Statistics. To minimize misclassification of case status, only cases verified by another internal (e.g., drug use) or external (record linkage) source were included.

### 2.6 Statistical Analysis

#### 2.6.1 Data Preparation

Because of the skewed distributions and the frequency structure of the reported dairy consumption, the study classified dairy consumption into low and high consumers for milk, yogurt, cheese, butter, and total dairy products. Milk consumption was classified into <1 serving day^−1^ and ≥2 servings day^−1^. Yogurt, cheese, and butter consumption were classified into <1 serving week^−1^ and ≥1 serving day^−1^. Total dairy consumption was classified into <1 serving day^−1^ and ≥3 servings day^−1^. The study derived the energy densities of milk and total dairy products per 2000 kcal day^−1^ and used the two variables as continuous.

Metabolomics and fatty acid data were log‐transformed, mean‐centered, and standardized to units of standard deviation (SD). Due to the low missingness (<3% of participants for any covariate), the study imputed missing information on covariates using single imputation.^[^ ^18^ ^]^

#### 2.6.2 Derivation of Models Classifying Dairy Consumption

In the derivation set regression models were developed, classifying the consumption of individual dairy types by 82 metabolites that were common to the two studies, Fenland and EPIC‐Norfolk (Figure 1). The study fitted an elastic‐net penalized logistic regression model regressing each of binary dairy variables on the 82 metabolites selected to derive estimates robust against high multicollinearity between the metabolites, with a low prediction error among common regression‐based methods.^[^ ^19^ ^]^

The study developed five regression models with different sets of covariates for each dairy type (Table S2, Supporting Information). The primary model included 82 metabolites controlling for the covariates (age, sex, test site, smoking status, physical activity, lipid‐lowering drugs, hormone replacement therapy [in women], and body mass index [BMI]).

The study created metabolite scores from the sum of the metabolite concentrations weighted by the coefficients estimated from elastic‐net regression. Metabolites with regression coefficients were selected outside the range of mean±2×SD of all the 82 coefficients. The scores derived based on selected metabolites were determined post hoc because the preliminary analysis including all the metabolites produced scores apparently sensitive to random noise because of the small magnitude of the regression coefficients of many metabolites.

#### 2.6.3 Internal and External Validation Analyses

Each metabolite score was tested for whether it could classify high and low consumers of each dairy type (subtypes and total) in the two validation datasets (Fenland and EPIC‐Norfolk) (Figure 1). The study fitted standard (no penalization) multivariable‐adjusted logistic regression models including each metabolite score as a single predictor, each of the binary dairy variables as a dependent variable, and potential confounders selected as above. As the primary metric for the ability to classify high and low dairy consumption, the area under the curve (AUC) was estimated. The net reclassification improvement (NRI) was also estimated. The likelihood ratio test was used to compare different nested logistic regression models.

The study further evaluated whether each metabolite score would improve the classification ability over and above the plasma phospholipid fatty acids OCSFAs and C16:1n7*t*, well‐recognized biomarkers of dairy fat intake.^[^ ^3^ ^]^ Three logistic regression models were fitted, with the same covariates as above, to classify dairy consumption (Table S2, Supporting Information). The independent variables were 1) OCSFAs, C16:1n7*t;* 2) the metabolite score; and 3) the metabolite score and the three fatty acids.

#### 2.6.4 Prospective Analysis for Incident T2D

Using metabolite scores classifying between low and high dairy consumption in the external validation, the associations between the metabolite scores and T2D incidence in the nested case‐cohort subset of the EPIC‐Norfolk Study were evaluated. Prentice‐weighted Cox proportional hazard models were fitted to estimate hazard ratios (HR) and their confidence intervals (CI). HR estimates were adjusted for age, sex, educational level, socio‐economic status, family history of T2D, smoking, physical activity, lipid‐lowering drugs, anti‐hypertensive drugs, hormone‐replacement therapy (in women), dietary supplement use, dietary variables (continuous variables of intakes of total energy, fruit, vegetables, cereals, red meat, processed meat, margarine, sweet snacks, sugar‐sweetened beverages, coffee, tea, and alcoholic beverages), and BMI. The proportional‐hazards assumption was tested with Schoenfeld residuals.

#### 2.6.5 Secondary and Post‐hoc Analyses

The study used two secondary approaches for the derivation of the metabolite scores. First, the initial internal validation set was used as a derivation set to also include the two OCSFAs, in addition to the 82 matched metabolites in the derivation set (internal absolute validity not possible to assess). Second, all 174 metabolites were included from the targeted platform of the derivation set (external validation not possible). As per the Transparent Reporting of a multivariable prediction model for Individual Prognosis Or Diagnosis guidelines,^[^ ^20^ ^]^ the study repeatedly analyzed the derivation set using models without penalization. The energy densities of milk and total dairy consumption were used as secondary outcomes in all analyses, for which R^2^ was estimated as a performance measure, modeling multivariable‐adjusted linear regression.

To assess the sensitivity of the primary results to the criterion to select top metabolites based on the mean ± 2×SD of 82 coefficients, the criterion to the mean ± 1×SD was changed. A score was also created, retaining all the metabolites. To assess the specificity of the metabolite scores to the dairy types, the study examined the associations of each score with consumption of the dairy types that the score was not derived for, and with 17 other food groups in the external validation set. Cox regression was performed for T2D using metabolite scores not significantly predicting dairy consumption in the external validation. The study conducted two post‐hoc analyses. First, because the two studies used different metabolomics platforms, the study assessed whether or not the metabolite variance‐covariance matrices were similar between the two studies using an asymptotic chi‐square test.^[^ ^21^ ^]^ Second, the performance of the scores was assessed, when the metabolite was removed from them which most consistently contributed the most to the scores.

Analyses were performed with Stata 14.2 (College Station, TX, USA, StataCorp LP, 2015) except for the development and validation of models, which were performed in Python 3.6.3 Jupyter Notebooks^[^ ^22^ ^]^ using the Scikit Learn module v0.19.1^[^ ^23^ ^]^ for elastic‐net regression and estimation of metrics and the Statsmodels v0.9.0 module for standard logistic and linear regression analyses.

## 3 Results

The distributions of socio‐demographic, lifestyle, and dietary factors were similar in the derivation and internal validation sets in the Fenland Study but with some variation between the Fenland Study and the external validation set from the EPIC Norfolk Study (**Table** **1**). For example, EPIC Norfolk included a 10‐year older population, with a larger proportion taking antihypertensive medication and hormone‐replacement therapy.

**Table 1:** Participant characteristics in the Fenland study (2005–2015) and the EPIC Norfolk diabetes case‐cohort study (1993–1997), the United Kingdom.

Characteristics^[Link]^, ^a)^	Fenland study				EPIC Norfolk study	
	Derivation		Internal validation^c)^		External validation^d)^	
	*n* = 6035		*n* = 4246		*n* = 825	
Age [years]	48.9	7.4	47.8	7.3	59.0	9.4
Sex, women [%]	53.2		54.5		58.1	
Educational level, medium [%]^a)^	45.9		45.9		38.8	
High [%]	34.4		33.7		13.9	
Smoking, former [%]^a)^	33.9		32.3		39.0	
Current [%]	11.7		12.8		13.0	
Physical activity						
Energy expenditure [kj kg^−1^ d^−1^]	53.7	22.1	54.4	22.4	Na^e)^	
Moderately active or active [%]^a)^	Na^e)^		Na^e)^		43.4	
BMI [kg m^−2^]	26.9	4.8	26.8	4.7	26.1	3.7
Lipid‐lowering drugs [%]^a)^	4.3		3.5		1.3	
Antihypertensive drugs [%]	7.2		7.2		34.2	
Hormone replacement therapy, % among women	2.9		2.8		19.2	
Dairy products^a)^						
Milk <1 serving d^−1^ [%]	38.8		37.8		22.3	
≥2 serving d^−1^ [%]	27.0		27.1		40.2	
Yogurt, <1 serving wk^−1^ [%]	35.6		36.6		48.7	
≥1 serving d^−1^ [%]	13.1		12.6		8.4	
Cheese, <1 serving wk^−1^ [%]	27.9		28.0		22.7	
≥1 serving d^−1^ [%]	8.1		9.0		8.6	
Butter, <1 serving wk^−1^ [%]	51.9		53.9		62.2	
≥1 serving d^−1^ [%]	17.8		18.3		18.4	
Total dairy products, <1 servings d^−1^	6.7		7.0		3.9	
≥3 servings d^−1^	39.1		39.4		44.1	
Milk, % energy	1.5	1.0	1.6	1.0	1.8	0.9
Total dairy products, % energy	3.0	1.4	3.0	1.4	3.1	1.4
Non‐dairy dietary factors						
Dietary supplements [%]	41.0		42.4		50.8	
Energy intake [kcal d^−1^]	1924	571	1939	579	2013	558
Fruits [g d^−1^]	240.6	203.3	244.8	198.7	248.8	197.8
Vegetables [g d^−1^]	258.1	143.2	253.4	135.5	239.2	118.2
Cereals [g d^−1^]	169.3	100.6	169.2	96.4	157.4	84.4
Red meat [g d^−1^]	74.2	46.7	72.9	48.4	63.4	46.0
Processed meat [g d^−1^]	31.7	26.9	32.3	29.3	29.2	24.3
Fish [g d^−1^]	42.9	33.4	43.2	35.4	38.1	25.5
Sugar‐sweetened beverages [g d^−1^]	43.2	95.1	39.9	84.7	35.0	73.6
Alcoholic beverages [g d^−1^]	151.2	249.5	149.7	238.1	248.8	202.6

EPIC, European Prospective Investigation into Cancer and Nutrition; T2D, type 2 diabetes.

^a)^ The mean and SD are presented for continuous variables and column percentages are presented for categorical variables. Categories for “low” or “no” status were omitted for education, physical activity, smoking, medications, and dietary supplement; and for mid‐categories of dairy consumption (neither low nor high);

^b)^ Missing values for each variable were < 3% in the Fenland Study and <2% in the EPIC Norfolk Study;

^c)^ The internal validation was the set that had been previously randomly selected for measurement of blood fatty acids;

^d)^ The characteristics of the random sub‐cohort from the EPIC case‐cohort study are presented (The longitudinal analysis evaluated 1440 participants in total, including 641 T2D incident cases);

^e)^ na, not applicable. Physical activity was objectively assessed in the Fenland study and expressed as a continuous variable for physical activity energy expenditure. Physical activity levels were assessed with a questionnaire in the EPIC Norfolk study and categorized into four categories.

### 3.1 Derivation, Internal, and External Validation of Metabolite Scores Classifying Dairy Consumption

**Figure 2:**
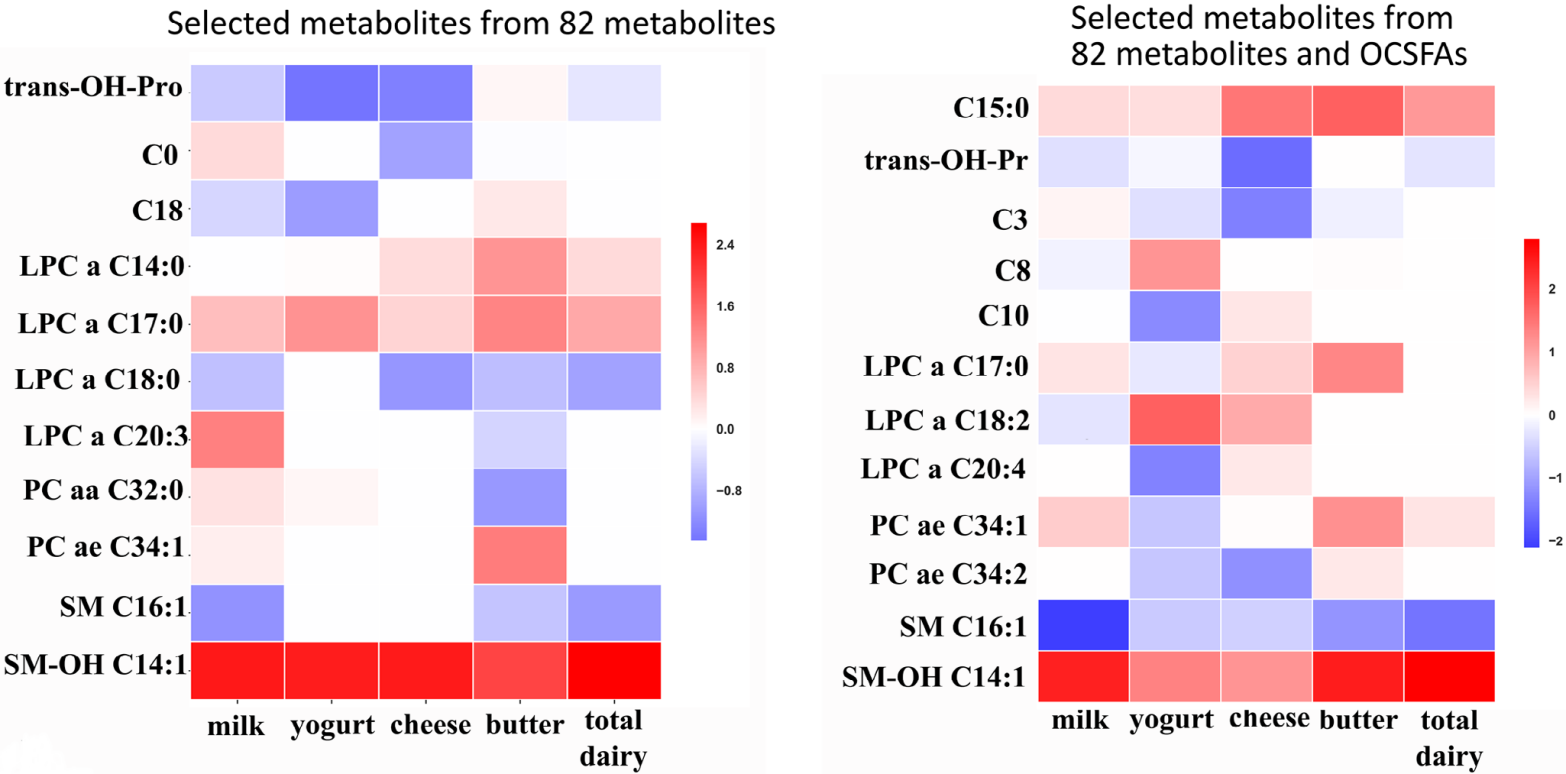
Relative strengths of associations of metabolites with consumption status of each dairy type: a derivation analysis in the Fenland Study (*n* = 6035). Two panels display the metabolites selected if each adjusted coefficient (per 1‐SD of each metabolite) was greater than mean+2×SD (red) or lower than mean‐2×SD (blue) calculated from all the coefficients of 82 metabolites (left) and 82 metabolites + 2 OCSFAs (right). The coefficients were adjusted for age, sex, test site, smoking status, physical activity, lipid lowering drugs, hormone‐replacement therapy, and BMI in the elastic‐net penalized regression models. Refer to Supplemental Methods for metabolite annotation. LPC, lyso‐phosphatidylcholines; OCSFAs, odd‐chain saturated fatty acids; PC, Phosphatidylcholines; Pro, proline; SM‐OH, hydroxyl‐sphingomyelins; SM‐OH C14:1: isobaric molecule of SM(d18:1/C15:0) and SM(d16:1/C17:0).

Among 82 metabolites available in the Fenland Study and the EPIC Norfolk Study, 11 metabolites were associated with consumption status of at least one dairy type (**Figure** **2**) after selecting metabolites with coefficients outside the mean ± 2×SD of the distribution. Hydroxy‐sphingomyelin (SM‐OH) C14:1 [representing its isobaric compounds SM(d18:1/C15:0) and SM(d16:1/C17:0)], appeared to classify consumption of all the dairy types similarly. Other signals classifying dairy consumption included SM C16:1 (milk and total dairy products), and lyso‐phosphatidylcholine a C17:0 (LPC a C17:0; for cheese, butter and total dairy products). *Cis*‐ and *trans*‐hydroxy proline (OH‐Pro) were negatively associated with yogurt, and *trans*‐OH‐Pro was negatively associated with cheese consumption. After adjustment for other dietary factors, of the top metabolite signals mentioned above, SM‐OH C14:1 [SM(d18:1/C15:0, d16:1/C17:0)] and LPC a C17:0 classified dairy consumption (data not shown).

In the internal validation set, adding the metabolite score to the model with non‐metabolite covariates (reference) increased the AUCs: from 0.59 to 0.80 for total dairy products, 0.59–0.72 for butter, 0.59–0.64 for milk, 0.67–0.73 for cheese, and 0.67–0.69 for yogurt (Figure S1, Tables S3 and S4, Supporting Information).

In the external validation using 11 metabolites, the metabolite scores better classified consumers of milk (AUC increased from 0.59 to 0.60, NRI = 0.25, *p* < 0.001). For butter and total dairy, AUC increased from 0.61 to 0.62 (NRI = 0.14, *p* = 0.02) and from 0.59 to 0.68 (NRI = 0.33, *p* = 0.008), respectively. The metabolite scores for yogurt and cheese did not show improvement (*p* = 0.09 and 0.22, respectively) (**Table** **2**).

**Table 2:** Classification ability for consumers and non‐consumers of different dairy products: external validation analysis of the metabolite scores derived from the Fenland Study and tested in the EPIC‐Norfolk Study, United Kingdom.

Components of the metabolite scores^a)^, ^b)^	Dairy type	*N*	*N* metabolites	AUC before/after adding the metabolite score^c)^		NRI	*p*_NRI_ ^d)^
				Before	After		
Selected metabolites^e)^	Milk	900	3	0.59	0.60	0.25	<0.001
	Yogurt	875	2	0.69	0.70	0.14	0.093
	Cheese	475	4	0.65	0.66	0.08	0.22
	Butter	1193	5	0.61	0.62	0.14	0.019
	Total dairy	687	4	0.59	0.68	0.33	0.008
Selected metabolites + fatty acids^e)^	Milk	362	2	0.61	0.65	0.25	0.012
	Yogurt	391	5	0.72	0.72	0.07	0.34
	Cheese	194	3	0.70	0.70	−0.06	0.63
	Butter	515	5	0.62	0.66	0.26	0.004
	Total dairy	282	3	0.61	0.77	0.76	0.001

AUC, area under the curve of a receiver operating characteristics; EPIC, European Prospective Investigation into Cancer and Nutrition; NRI, net reclassification improvement.

^a)^ All the statistics were adjusted for socio‐demographic and lifestyle factors include age, sex, test site, smoking status, physical activity, lipid lowering drugs, hormone‐replacement therapy, and BMI;

^b)^ The set of metabolites used in the analysis to derive the metabolite scores in the Fenland Study;

^c)^ “Before” refers to the model including covariates only; and “after”, the model including covariates and the metabolite score;

^d)^ *p* values for the metabolite score for the classification of consumers and non‐consumers;

^e)^ Selected metabolites from 82 metabolites or from 82 metabolites + fatty acids if the regression coefficients were outside the mean±2×SD.

### 3.2 Utility of the Metabolite Scores over the Use of Fatty Acids as Dietary Markers

OCSFAs and C16:1n7*t* classified high dairy consumers (Table S4, Supporting Information). Further addition of each metabolite score increased the classification ability in the internal validation (Tables S3 and S4, Supporting Information). In the external validation, derivations of metabolite scores with and without OCSFAs showed similar performance to the model without metabolites resulting in increases in the AUCs mainly for butter and total dairy products (Table 2). Further addition of the metabolite score to the models increased the classification ability in the internal validation, but not in the external validation (Table S3 and S4, Supporting Information).

### 3.3 Associations of Metabolite Scores Classifying Dairy Consumption with T2D Incidence

During 16 360 person‐years of follow‐up, each of the metabolite scores for milk, butter, and total dairy was inversely associated with T2D incidence (**Table** **3**). Multivariable‐adjusted models (not adjusted for BMI) showed a 29% lower T2D incidence [hazard ratio (HR) = 0.71, 95% confidence interval (CI): 0.65, 0.77] per 1 SD of the metabolite score classifying milk consumption. One SD of the metabolite scores for butter and total dairy consumption was associated with 38% [0.62 (0.57, 0.68)] and 34% [0.66 (0.60, 0.72)] lower T2D incidence, respectively (Table 3). After adjustment for BMI and then OCSFAs, the associations for the metabolite scores remained significant (Table 3). By contrast, these negative associations were not seen for the metabolite scores for yogurt and cheese consumption, which did not show evidence of external validity as above mentioned (Table S5, Supporting Information).

**Table 3:** Associations of metabolite scores classifying dairy consumption with T2D incidence: nested case‐cohort analysis of the EPIC Norfolk study.

Metabolite score for each dairy type^a)^, ^b)^	Model	HR	95% CI
Milk	Socio‐demographic + family history of T2D^c)^	0.67	0.63, 0.73
	+Smoking, physical activity, drugs^d)^	0.67	0.62, 0.73
	+ Diet^e)^	0.71	0.65, 0.77
	+ BMI	0.75	0.69, 0.82
	+ OCSFAs	0.71	0.61, 0.83
Butter	Socio‐demographic + family history of T2D^c)^	0.59	0.54, 0.64
	+Smoking, physical activity, drugs^d)^	0.62	0.57, 0.67
	+ Diet^e)^	0.62	0.57, 0.68
	+ BMI	0.60	0.54, 0.65
	+ OCSFAs	0.53	0.46, 0.62
Total dairy	Socio‐demographic + family history of T2D^c)^	0.63	0.58, 0.67
	+Smoking, physical activity, drugs^d)^	0.64	0.59, 0.69
	+ Diet^e)^	0.66	0.60, 0.72
	+ BMI	0.74	0.67, 0.80
	+ OCSFAs	0.68	0.58, 0.79

CI, confidence interval; EPIC, European Prospective Investigation into Cancer and Nutrition; HR, hazard ratio; OCSFA, odd‐chain saturated fatty acids; T2D, type 2 diabetes.

^a)^ Based on analysis of 641 cases of T2D and 16 360 person‐years of follow‐up, HRs and 95% CIs were estimated per one SD difference in each metabolite score. All the associations were significant (*p* < 0.001) except for the metabolite score for milk, which included all the 82 metabolites after adjustment for BMI;

^b)^ Metabolite scores generated from the total sample of the incident diabetes case‐cohort study (*n* = 1440). Selected metabolites were the top metabolites defined as those with absolute values of elastic net coefficients >mean+2×SD;

^c)^ Adjusted for age (continuous in years), sex, educational level (low, medium, high), socio‐economic status (low, medium, high), and family history of T2D (yes, no);

^d)^ Additionally adjusted for smoking (never, former, current), physical activity (inactive, moderately inactive, moderately active, active), lipid‐lowering drugs (yes, no), anti‐hypertensive drugs (yes, no), hormone‐replacement therapy (yes, no, men);

^e)^ Additionally adjusted for total energy intake (kcal day^−1^), dietary supplement use (yes, no), consumption (g day^−1^) of fruit, vegetables, total cereals, red meat, processed meat, fish, margarine, sweet snacks, sugar‐sweetened beverages, coffee, tea, alcoholic beverages.

### 3.4 Secondary Analyses

When we repeated the derivation analyses in Fenland including both 82 metabolites and OCSFAs, 12 metabolites appeared to classify consumers of one or more dairy types (C15:0, two acylcarnitines, five PCs, and two SMs) (Figure 2, Figures S2–S4, Supporting Information). Internal validation of the metabolite scores for milk and total dairy products as continuous variables and when using a total of 174 metabolites in the Fenland Study, gave similar results. The variance‐covariance patterns of 82 metabolites or the top 11 metabolites selected for one or more of the metabolite scores differed significantly between the two cohorts (*p* for the matrix homogeneity <0.0001; Figure S5, Supporting Information). When removing SM‐OH C14:1 [SM(d18:1/C15:0, d16:1/C17:0)], which was the most consistent top metabolite, from the scores, there was a smaller improvement in the classification ability of the scores, but still significant for butter and total dairy products (Table S6, Supporting Information).

When selecting metabolites if they had coefficients outside mean±1×SD, a smaller improvement in the classification ability of the scores was observed compared to the main scores (Table S6, Supporting Information). Each metabolite score showed null or heterogeneous associations with consumption of dietary components in EPIC‐Norfolk (Table S7, Supporting Information). Secondary analyses with different analytic strategies to derive metabolite scores yielded consistent associations with incident T2D overall with those from the primary analysis (Table S8, Supporting Information).

## 4 Discussion

In analyses conducted using two independent studies we identified sets of metabolites to classify physiological response between low and high habitual consumption of milk, butter, and total dairy products. These metabolite scores with evidence of external validity were associated with lower T2D incidence.

### 4.1 1. Findings in Context of Other Evidence

The associations between dairy‐related metabolites and T2D that we observed were consistent with those from meta‐analyses evaluating self‐reported consumption of butter^[^ ^2^ ^]^ and total dairy products.^[^ ^1^ ^]^ Meta‐analyses of milk reported null associations with T2D risk,^[^ ^1^ ^]^ which might mean that biological pathways other than the pathway potentially reflected in our results might differentially link milk to T2D.

The findings for metabolite scores classifying milk, butter and total dairy consumption were consistent with previous evidence of inverse associations of SM‐OH C14:1,^[^ ^24^, ^25^ ^]^ LPC a C17:0,^[^ ^24^, ^25^, ^26^ ^]^ PC ae C34:1,^[^ ^24^ ^]^ and OCSFAs,^[^ ^27^ ^]^ with T2D risk or other glycemic outcomes. A study with similar methodology to ours, but a different set of metabolites, observed inverse associations of metabolite scores predicting total dairy, milk, yoghurt, and cheese consumption with T2D risk in their derivation set, but only for total dairy and milk in their external validation set,^[^ ^5^ ^]^ partly consistent with our findings. Notably, the external validity in that study was also weak for yoghurt and cheese^[^ ^5^ ^]^ consistent with our finding. Heterogeneity and variability across fermented dairy products over time may have resulted in weak comparability in the metabolome between the two independent cohorts that collected dietary data more than 10 years apart on average.^[^ ^5^ ^]^

Of the metabolites we identified for classification of dairy consumers, SM‐OH C14:1 [SM(d18:1/C15:0, d16:1/C17:0)]^[^ ^6^, ^28^ ^]^ was observed to be associated with dairy consumption in two other cohorts providing support that this metabolite may serve as a potential dairy biomarker. When we included the OCSFAs in the metabolite set of our derivation analysis, C15:0 was one of the top signals for the classification of butter consumption, which confirms previous evidence.^[^ ^3^ ^]^ We did not identify C17:0^[^ ^29^ ^]^ as a dairy biomarker when we evaluated it together with the metabolites. Failure to do so may have been due to high correlations between C17:0 and other metabolites, e.g., LPC a C17:0, which predicted high‐fat dairy consumption overall in a randomized controlled trial.^[^ ^30^ ^]^ As demonstrated by Münger et al., only a few novel metabolites have been identified for dairy products with insufficient validation, but studies that have explored this, including randomized controlled trials, have identified OCSFAs from the phospholipids fraction or as intact phospholipid or sphingomyelins.^[^ ^31^ ^]^ Future work will be essential to profile lipid species, such as phospholipids, sphingomyelins, and cholesteryl esters, which contain OCSFAs and ruminant trans‐FAs.

### 4.2 Biological Interpretation

From our metabolomics profiling, mainly lipids were identified to classify physiological response between low and high dairy consumption. Among such metabolites, SM‐OH C14:1 appeared to robustly classify different dairy products with different analytical approaches. As mentioned, this lipid is an isobaric molecule of SM(d18:1/C15:0) and SM(d16:1/C17:0), which contain OCSFAs. This means that these molecules have the same mass, and they would need a more sensitive platform to distinguish between them. Presence of such isobaric compounds, as well as high correlations between different food groups in our study highlight the challenges in identifying molecules specific to different dairy types. LPC a C17:0 was also identified as one of the top metabolite signals for classification. This metabolite contains the OCSFA C17:0, a candidate biomarker of dairy fat. A similar explanation about OCSFAs could be given for the observed associations of LPC 14:0 (C14:0) and PC ae 34:1 (C14:0 and C15:0). The inverse associations observed for some metabolites, e.g. hydroxy‐proline isomers, might reflect more indirect pathways and are thus more challenging to interpret.

The association of circulating OCSFAs and C16:1n7t with lower incidence of T2D has been previously reported.^[^ ^27^ ^]^ Self‐reported butter consumption has also been previously associated with lower T2D risk.^[^ ^2^ ^]^ Thus, the study of the potential effect of dairy fat on the T2D risk and its biological mechanisms are worthy of future investigation.

### 4.3 Strengths and Limitations

The key strength of our study is the development of metabolite scores with multiple metabolites classifying dairy consumption in analyses including both internal and external validation in two independent large population‐based studies. Despite the 10‐year difference in the participant recruitment, we found evidence of external validity for scores of total dairy, milk and butter (temporal generalisability), but not yogurt and cheese. Although a previous study followed similar methodology to ours,^[^ ^5^ ^]^ our availability of plasma phospholipid fatty acids and metabolomics additionally allowed us to compare the known biomarkers and new data‐driven metabolite biomarkers. However, we had a limited ability to derive biomarkers for variation of specific dairy consumption because of the inherent correlations between dairy consumption, consumption of other foods, and behavioral factors. Rather, we should interpret that we detected metabolomics markers for habitual dairy consumption within overall diets and in the context of other behavioral factors. Although the correlation matrices of top metabolites between the two studies significantly differed, we were still able to find evidence of external validity in the metabolite scores for milk, butter, and total dairy products. The metabolites we evaluated may not fully capture the metabolome in the blood or in other potentially relevant tissues. Finally, our studies were based largely on populations of white British background, so our results may not be generalizable to other ethnic groups or populations with different dairy consumption patterns, e.g., populations with high lactose intolerance.

## 5 Conclusion

In this study, we identified metabolite scores discriminating between physiological response to high and low consumers of milk, butter, and total dairy products with internal and external validity. Analysis using the metabolite scores supported the association of those dairy products with a lower T2D risk. In contrast, this study did not identify any sets of metabolites that classify yogurt or cheese consumption with external validity in our study. Our methodological approach and findings should stimulate further observational studies and trials including markers from other assays, biological samples, e.g., gut microbiome in diverse populations to better understand how consumption of a certain dietary product may influence cardio‐metabolic risk.

## Conflict of Interest

The authors declare no conflict of interest.

## Author Contributions

F.I. and N.G.F. contributed equally to this work. N.J.W., C.L., S.B., S.J.G., K.T.K., and N.G.F. designed research; N.G.F., F.I., and E.T. conceived the research question; A.K. and J.G. designed and managed laboratory analyses; A.K. managed and provided laboratory data; E.T. and F.I. analyzed data; E.T., F.I., and N.G.F. prepared the initial draft of the manuscript which all authors contributed to improve, and all authors read and approved the final version. E.T. and N.G.F. had primary responsibility for final content.

## Supporting information

Supporting Information.

## Data Availability

The datasets generated and analysed during the current study are available at request via the MRC Epidemiology Unit (http://www.mrc‐epid.cam.ac.uk/research/data‐sharing/).
