# Supplementary material for: Plasma Metabolites Related to the Consumption of Different Types of Dairy Products and Their Association with New‐Onset Type 2 Diabetes: Analyses in the Fenland and EPIC‐Norfolk Studies, United Kingdom
Source: Mol Nutr Food Res. 2023 Dec 6;68(1):2300154. doi: 10.1002/mnfr.202300154 (PMC10909549; doi:10.1002/mnfr.202300154)
Supplement: Supplementary file 1 — Supporting Information. [file MNFR-68-2300154-s001.pdf]

Supplementary material

Plasma metabolites related to consumption of different types of dairy products and their association with new-onset type 2 diabetes: analyses in the Fenland and EPIC-Norfolk studies, United Kingdom

Eirini Trichia PhD[1] + co-authors

Contents

Supplemental methods 3

Supplemental Table 1. Metabolite matching between the targeted metabolomics platform in the Fenland study and the untargeted platform in the EPIC Norfolk diabetes case-cohort study 7

Supplemental Table 2. Covariates used in different models for the different analyses in the Fenland and EPIC-Norfolk studies 10

Supplemental Table 3. Likelihood ratio (LR) tests and net reclassification improvement (NRI) for the models with or without the fatty acids* and the metabolite score in the fatty acid set of the Fenland study for internal validation (n=4,246) and EPIC-Norfolk study for external validation (n=592) 11

Supplemental Table 4. Areas under the curve of the logistic models* with or without the fatty acids C15:0, C17:0, C16:1n7t and the metabolite score (with selected metabolites †) for total and types of dairy products in the internal validation set of the Fenland study (n=4,246) and the external validation set of the EPIC-Norfolk diabetes case-cohort study (n=592). 12

Supplemental Table 5. Hazard ratios of T2D per 1 SD of metabolite scores not significantly associated with dairy consumption after 16,360 person-years of follow-up (641 cases of T2D) in the incident diabetes case-cohort study within the EPIC Norfolk study 13

Supplemental Table 6. Classification ability of the metabolite scores for consumption of different dairy products: a secondary external validation analysis of the metabolite scores derived from the Fenland Study and tested in the EPIC-Norfolk Study, United Kingdom* 14

Supplemental Table 7. Associations of metabolite scores including selected top metabolites classifying each of total and types of dairy consumption with other dairy products and food groups in the EPIC Norfolk case-cohort study 15

Supplemental Table 8. Hazard ratios of T2D per 1 SD of metabolite scores classifying dairy consumption in the incident diabetes case-cohort study within the EPIC Norfolk study* 16

Supplemental Figures 17

Supplemental Figure 1. Receiver operating characteristic (ROC) curves and corresponding AUCs for prediction models of metabolite scores reflecting (A) milk, (B) yogurt, (C) cheese, (D) butter and (E) total dairy consumption in the internal validation set (n=4,246). 17

Supplemental Figure 2. Strength of associations of metabolites, socio-demographic and lifestyle covariates with consumption levels of total and types of dairy products in the derivation set of the Fenland study with the 82 metabolites matched with those in the diabetes case-cohort of the EPIC Norfolk study. 19

Supplemental Figure 3. Strength of associations of metabolites, socio-demographic and lifestyle covariates with consumption levels of total and types of dairy products in the derivation set of the Fenland study with the 82 metabolites matched with those in the diabetes case-cohort of the EPIC Norfolk study and the OCSFAs. 21

Supplemental Figure 4. Strength of associations of metabolites, socio-demographic and lifestyle covariates with consumption levels of total and types of dairy products in the derivation set of the Fenland study with the whole set of 174 metabolites. 25

Supplemental Figure 5. Correlation matrices of the selected top metabolites associated with at least one of each of milk, yogurt, cheese, butter or total dairy consumption in the Fenland and EPIC Norfolk studies. 26

Supplemental methods

Metabolite and fatty acid samples processing

Sample derivation and internal validation sets – the Fenland study

Blood samples were collected into lithium heparin coated tubes and spun to separate the plasma. Samples were stored in -80ºC in freezers until analyzed. Before the analysis, the samples were removed from the freezer, placed on roller mixers at 30 rpm to thaw for 15-20 minutes and centrifuged for 1 minute at 2000 rpm. Calibration standards and internal standards were also briefly centrifuged and quality control (QC) samples were centrifuged for 5 minutes at 2750 rpm. All vials were then shaken for 15 minutes at 1200 rpm and vortexed and then transferred to 96-well plates with rows A-H and columns 1-12. Derivatization agent (50 µl) was added to all the wells. The internal standards (10 µl) were added to all wells for quantification apart from A1, which belonged to the blank (deionized water with phosphate buffer saline). Positions B2-D2 contained the zero samples (blank samples with internal standards). Positions E1-C2 contained the calibration samples, positions D2-F2 contained the commercial QC samples, positions G2 and H12 contained the pooled QC samples and positions H3-G12 contained the study samples. The sample processing was done on a Hamilton STAR liquid handling station (Hamilton Robotics Ltd, Birmingham, UK). Targeted metabolomic profiling of 10,684 blood plasma samples was performed with a commercial kit (Absolute IDQ p180 kit, BIOCRATES Life Sciences AG, Innsbruck, Austria). Flow injection analysis (FIA) MS (AB SCIEX 5500 Qtrap mass spectrometer, Sciex Ltd, Warrington, UK), isocratic with methanol, in positive ionization mode was performed for lipids and acylcarnitines. FIA-MS in negative ionisation mode was performed for hexose. Ultra-performance liquid chromatography MS (UPLC-MS; Waters ltd, Manchester, UK coupled to ABSciex 5500 Qtrap mass spectrometer, Sciex Ltd, Warrington, UK) was performed for amino acids and biogenic amines with a 5-minute gradient elution starting with 100% water and increasing to 95% acetonitrile and 0.2% formic acid over a Waters Acquity UPLC BEH C18 column (2.1 x 50 mm, 1.7 µm). Amino acids and biogenic amines were quantified and expressed in an absolute scale, whereas lipids and hexose were semi-quantified and expressed in a relative scale.

The raw metabolomics data were processed with the MetIDQ software (provided with the kit). Then the data went through a check for outliers, non-detects, peak picking, normalization with the use of the QC samples (metabolite values were divided by the ratio of

the mean value of the QC samples within a plate to the mean value of the samples across all the plates) and finally batch correction. During batch correction, zero values were replaced with numbers randomly selected from a uniform distribution between 0.1 and a minimum observed within batch.

External validation set – the EPIC Norfolk study

From sample collection to sample preparation to be shipped to Metabolon, Inc., blood samples were stored in the gas phase of liquid nitrogen (-175ºC). From sample preparation to shipment to Metabolon, samples were stored in -70ºC. Upon receipt of the samples by Metabolon, they were stored in -80ºC until one day before the analysis and overnight under nitrogen the night before the analysis. Deproteinisation was done with methanol, under vigorous shaking for 2 minutes (Glen Mills GenoGrinder 2,000). The samples were then centrifuged and placed on a TurboVap (Zymark) to remove the organic solvent. The order of the study samples on the plates was randomized and QC samples (two commercial QC standards and 4 pooled QC samples) were evenly distributed across the study samples. A blank sample and internal standards were also used. Untargeted metabolomic profiling was performed in 1,503 blood citrated plasma samples (DiscoveryHD4® platform, Metabolon, Inc.; Waters ACQUITY UPLC, Thermo Scientific Q-Exactive spectrometer with a heated electrospray ionization (HESI-II) source and Orbitrap mass analyzer with 35,000 mass resolution). Samples were divided into five fractions of which two were used for reverse phase UPLC MS/MS with positive ion mode ESI (mobile phases with 0.1% formic acid in water and 0.1% formic acid in methanol), one was used for reverse phase UPLC MS/MS with negative ion mode ESI (mobile phases with 6.5 mM ammonium bicarbonate in water, pH=8 and 6.5 mM ammonium bicarbonate in 95% methanol and 5% water), one was used for hydrophilic interaction liquid chromatography (HILIC) UPLC MS/ MS with negative ion mode ESI (mobile phases with 10mM ammonium formate in 15% water, 5% methanol, 80% acetonitrile and 10mM ammonium formate in 50% water and 50% acetonitrile) and one was kept as a backup. The UPLC column used was a Water BEH C18 (2.1 x 100 mm, 1.7 µm) at 40ºC and the HILIC column was a Waters BEH Amide (2.1 x 50 mm, 1.7 µm) at 40ºC. Gradient elution with water, methanol, 0.05% pentafluoropropionic anhydrate and 0.1% formic acid was used in all the columns. Metabolite identification was based on the retention index, accurate mass matched to the Metabolon internal library of metabolites within a range of ±10 ppm and the standards used. The raw metabolomics data were processed and went

through normalization by ``block correction'' i.e. setting median to 1 and adjusting each data point proportionately and QC checks with in-house software from Metabolon, Inc.

Metabolite annotation

For all the lipids, ``Cx:y'' indicates x carbon atoms and y double bonds. For the acylcarnitines, x denotes the number of carbon atoms of the carboxylic acid, which is esterified with carnitine. So carnitine is denoted as C0. For PCs there is the additional indication of ``aa'' when it contains two fatty acids bound through an ester linkage or ``ae'', when one of the two fatty acids were bound through an ether bond. So a PC might be denoted as PC aa Cx:y or as PC ae Cx:y. For LPCs, ``a'' denotes the presence of a fatty acid, so then this becomes LPC aa Cx:y or LPC ae Cx:y. SMs are denoted as SM Cx:y or SM-OH Cx:y if it is a hydroxy-SM.

Fatty acids assay

Phospholipid fractions were isolated from total plasma lipids with solid-phase extraction. Then free fatty acids were extracted with hydrolysis and derivatization and methylated to form fatty acid methyl-esters. These esters were then used in gas chromatography (7890N; Agilent Technologies) with a 30 m capillary column with a diameter of 0.25 mm. Sample was processed with sequential multipurpose sampler systems (Gerstel GmbH & Co. KG, Mulheim an der Ruhr, Germany). One blank sample and two QC samples were used in all the batches: one from human plasma pooled from the study samples and one from horse plasma. Standards of all fatty acids were used for the gas chromatography calibration. Study and QC samples were stored in -80ºC before analysis. Fatty acids were identified by comparing their retention times with those of the standards.

Approximate metabolite matching between the targeted and untargeted metabolomics

For the external validation, the metabolites between the targeted and untargeted metabolomics platforms were matched. Exact matching was done for 21 out of 21 amino acids, 10 out of 13 biogenic amines and 15 out of 40 acylcarnitines (**Supplemental Table 1**). For phosphatidylcholines (PCs), lyso-phosphatidylcholines (LPCs) and sphingomyelins (SMs), the FIA-MS/MS did not allow for the identification of the fatty acids in these lipids. Thus, the lipids reported from the targeted metabolomics platform are the sum of all their isobaric (same weight within a range of ±0.5 Da) and isomeric (same number of atoms, but different structure) compounds. Likewise for hexose sugars. The untargeted metabolomics platform identified specific isobaric/isomeric compounds for some lipids, while for others, it

also identified the sum of compounds, but with a higher precision. Lipids were matched approximately based on the number of carbon atoms, the number of double bonds and the molecular mass. After this process, approximate matching was possible for 9 out of 14 LPCs, 17 out of 74 PCs, 8 out of 11 SMs, and hexose (**Supplemental Table 1**).

Supplemental Results

Supplemental Table 1. Metabolite matching between the targeted metabolomics platform in the Fenland study and the untargeted platform in the EPIC Norfolk diabetes case-cohort study

Metabolite class	Metabolite name (targeted)	Metabolite abbreviation (targeted)	Matched metabolite name (untargeted)	Matched metabolite abbreviation (untargeted)
**Matched approximately between the two platforms**[a]				
Carnitines	Carnitine	C0	carnitine	C0
	Acetylcarnitine	C2	acetylcarnitine	C2
	Propionylcarnitine	C3	propionylcarnitine	C3
	Butyrylcarnitine	C4	butyrylcarnitine	C4
	Hydroxybutyrylcarnitine	C3DCC4OH	hydroxybutyrylcarnitine	C3DCMC5OH
	Tiglylcarnitine	C5:1	tiglyl carnitine	C5:1
	Methylglutarylcarnitine	C5MDC	3-methylglutarylcarnitine (2)	C5MDC
	Hexanoylcarnitine	C6C41DC	hexanoylcarnitine	C6C41DC
	Octanoylcarnitine	C8	octanoylcarnitine	C8
	Decanoylcarnitine	C10	decanoylcarnitine	C10
	Dodecanoylcarnitine	C12	laurylcarnitine	C12
	Tetradecanoylcarnitine	C14	myristoylcarnitine	C14
	Tetradecenoylcarnitine	C14:1	myristoleoylcarnitine	C14:1
	Hexadecanoylcarnitine	C16	palmitoylcarnitine	C16
	Octadecanoylcarnitine	C18	stearoylcarnitine	C18
	Octadecadienylcarnitine	C18:2	linoleoylcarnitine	C18:2
Lyso- phosphatidylcholines (LPC)	LPC acyl C140	LPC a C14:0	1-myristoyl-glycero-phosphocholine (GPC) (14:0)	lysoPCaC140
	LPC acyl C161	LPC a C16:1	1-palmitoleoyl-GPC (16:1)	lysoPCaC161
	LPC acyl C170	LPC a C17:0	1-margaroyl-GPC (17:0)	lysoPCaC170
	LPC acyl C180	LPC a C18:0	1-stearoyl-GPC (18:0)	lysoPCaC180
	LPC acyl C182	LPC a C18:2	1-linoleoyl-GPC (18:2)	lysoPCaC182
	LPC acyl C204	LPC a C20:4	1-arachidonoyl-GPC (20:4)	lysoPCaC204
	LPC acyl C160	LPC a C16:0	1-palmitoyl-GPC (16:0)	lysoPCaC160
	LPC acyl C181	LPC a C18:1	1-oleoyl-GPC (18:1)	lysoPCaC181
	LPC acyl C203	LPC a C20:3	1-eicosatrienoyl-GPC (20:3)	lysoPCaC203
Phosphatidylcholines (PC)	PC diacyl C300	PC aa C30:0	1-myristoyl-2-palmitoyl-GPC (14:0/16:0)	PC(14:0/16:0)
	PC diacyl C320	PC aa C32:0	1,2-dipalmitoyl-GPC (16:0/16:0)	PC(16:0/16:0)
	PC diacyl C321	PC aa C32:1	1-palmitoyl-2-palmitoleoyl-GPC (16:0/16:1)	PC(16:0/16:1)
	PC diacyl C322	PC aa C32:2	1,2-dipalmitoleoyl-GPC (16:1/16:1)	PC(16:1/16:1)

Supplemental Table 1. Metabolite matching between the targeted metabolomics platform in the Fenland study and the untargeted platform in the EPIC Norfolk diabetes case-cohort study

**Metabolite class**	**Metabolite name (targeted)**	**Metabolite abbreviation (targeted)**	**Matched metabolite name (untargeted)**	**Matched metabolite abbreviation (untargeted)**
	PC diacyl C341	PC aa C34:1	1-palmitoyl-2-oleoyl-GPC (16:0/18:1)	PC (16:0/18:1)
	PC diacyl C342	PC aa C34:2	1-palmitoyl-2-linoleoyl-GPC (16:0/18:2)	PC(16:0/18:2)
	PC diacyl C361	PC aa C36:1	1-stearoyl-2-oleoyl-GPC (18:0/18:1)	PC(18:0/18:1)
	PC diacyl C362	PC aa C36:2	1-stearoyl-2-linoleoyl-GPC (18:0/18:2)	PC(18:0/18:2)
	PC diacyl C363 †	PC aa C36:3	1-palmitoyl-2-dihomo-linolenoyl-GPC (16:0/20:3)	PC (16:0/20:3)
			1-oleoyl-2-linoleoyl-GPC (18:1/18:2)	PC(18:1/18:2)
	PC diacyl C364	PC aa C36:4	1-palmitoyl-2-arachidonoyl-GPC (16:0/20:4)	PC(16:0/20:4)
	PC diacyl C365	PC aa C36:5	1-palmitoyl-2-eicosapentaenoyl-GPC (16:0/20:5)	PC(16:0/20:5)
	PC diacyl C384	PC aa C38:4	1-stearoyl-2-arachidonoyl-GPC (18:0/20:4)	PC(18:0/20:4)
	PC diacyl C385 †	PC aa C38:5	1-palmitoyl-2-docosapentaenoyl-GPC (16:0/22:5n6)	PC(16:0/22:5n6)
			1-palmitoyl-2-docosapentaenoyl-GPC (16:0/22:5n3)	PC(16:0/22:5n3)
	PC acyl-alkyl C341	PC ae C34:1	1-pentadecanoyl-2-oleoyl-GPC (15:0/18:1)	PC(15:0/18:1)
	PC acyl-alkyl C342	PC ae C34:2	1-pentadecanoyl-2-linoleoyl-GPC (15:0/18:2)	PC(15:0/18:2)
	PC acyl-alkyl C361	PC ae C36:1	1-margaroyl-2-oleoyl-GPC (17:0/18:1)	PC(17:0/18:1)
	PC acyl-alkyl C362	PC ae C36:2	1-margaroyl-2-linoleoyl-GPC (17:0/18:2)	PC(17:0/18:2)
Sphingomyelins (SM)	SM C160	SM C16:0	palmitoyl sphingomyelin (d18:1/16:0)	SM(d18:1/16:0)
	SM C161	SM C16:1	sphingomyelin (d18:2/16:0, d18:1/16:1)	SM(d18:2/16:0, d18:1/16:1)
	SM C180	SM C18:0	stearoyl sphingomyelin (d18:1/18:0)	SM(d18:1/18:0)
	SM C181	SM C18:1	sphingomyelin (d18:1/18:1, d18:2/18:0)	SM(d18:1/18:1, d18:2/18:0)
	SM C241	SM C24:1	sphingomyelin (d18:1/24:1, d18:2/24:0)	SM(d18:1/24:1, d18:2/24:0)
	hydroxy SM C141	SM-OH C14:1	sphingomyelin (d18:1/15:0, d16:1/17:0)	SM(d18:1/15:0, d16:1/17:0)
	hydroxy SM C221	SM-OH C22:1	tricosanoyl sphingomyelin (d18:1/23:0)	SM(d18:1/23:0)
	hydroxy SM C222	SM -OH C22:2	sphingomyelin (d18:2/23:0, d18:1/23:1, d17:1/24:1)	SM(d18:2/23:0, d18:1/23:1, d17:1/24:1)
Hexose	Hexose [b]	Hexose	mannose	mannose
			fructose	fructose
			glucose	glucose

Matched exactly between two platforms[a,c]

Amino acids	Alanine	Ala	Glycine	Gly	Phenylalanine	Phe
	Arginine	Arg	Histidine	His	Proline	Pro
	Asparagine	Asn	Isoleucine	Ile	Serine	Ser

	Aspartate	Asp	Leucine	Leu	Threonine	Thr
	Citrulline	Cit	Lysine	Lys	Tryptophan	Trp
	Glutamine	Gln	Methionine	Met	Tyrosine	Tyr
	Glutamate	Glu	Ornithine	Orn	Valine	Val
Biogenic amines	Acetylornithine	AcOrn	Kynurenine	Kynurenine	Sarcosine	Sarcosine
	Symmetric dimethylarginine	SDMA	Methioninesulfoxide	Met-SO	Serotonin	Serotonin
	alpha-Aminoapidic acid	alpha-AAA	trans-hydroxyproline	t4OHPro	Taurine	Taurine
	Creatinine	Creatinine				

a) Approximately matched when a molecule from the targeted set was part of the sum of molecules in the untargeted set. The term exact matching refers to matching of the same molecule, although all the molecules in the untargeted platform are expressed in a relative scale, but amino acids and biogenic amines are expressed in an absolute scale in the targeted platform. The following molecules (n=92 in total) in the targeted metabolomics were not matched with any molecules in the untargeted metabolomics: Phenylethylamine, cis-hydroxyproline, spermidine, carnitine (C3:1, C3-OH, C4:1, C5, glutaconyl-C, glytaryl-C, hydroxyvaleryl-C, C6:1, pimelyl-C, C9, C10:1, C10:2, dodecanedioyl-C, C12:1, C14:1-OH, C14:2m C14:2-OH, C16:1, C16:1-OH, C16:2, C16:2-OH, C16-OH, C18:1, C18:1-OH), lysophosphatidylcholines (C26:1, C24:0, C26:0, C28:0, C28:1), phosphatidylcholine diacyls (C24:0, C26:0, C28:1, C32:3, C34:3, C34:4, C36:0, C36:6, C38:0, C38:1, C38:3, C38:6, C40s and 42s), phosphatidylcholine acyl-alkyls (C30s, C32s, C34:0, C34:3, C36:0, C36:3, C36:4, C36:5, C38s, C40s, C42s, C44s), sphingomyelins (C20:2, C24:0, C16:1-OH)

b) When molecules from the targeted set were matched with more than one molecules from the untargeted set, the sum of the molecules from the untargeted set was used.

EPIC: European Investigation into Cancer and Nutrition

c) Amino acids and biogenic amines were assayed in the both platforms with the same names and abbreviations, except for dimethylarginine (only SDMA in the targeted metabolomics, but both SDMA and asymmetric DMA in the untargeted metabolomics). Thus, those metabolite names and abbreviations are listed as they are.

Supplemental Table 2. Covariates used in different models for the different analyses in the Fenland and EPIC-Norfolk studies

Model	Covariates
**Derivation and validation of models**	
Model 0	age, sex, test site, physical activity, smoking status, lipid-lowering drugs, hormone-replacement therapy, BMI
Model 1	Metabolites
Model 2	Model 1 + model 0
Model 2a	Model 2 + educational level, occupational status, anti-hypertensive drugs
Model 3	Model 2 + total energy intake, fruit, vegetables, cereals, red meat, processed meat, fish, margarine, sweet snacks, sugar-sweetened beverages, caffeinated coffee, tea, alcoholic beverages, dietary supplements
**Utility of metabolite scores for the classification of dairy consumption over the C15:0, C17:0, and C16:1n7t fatty acids**	
Model 0	age, sex, test site, physical activity, smoking status, lipid-lowering drugs, hormone-replacement therapy, BMI
Model 1	C15:0, C17:0, C16:1n7t fatty acids
Model 1a	Metabolite score
Model 2	Model 1 + model 0
Model 2a	Model 1a + Model 0
Model 2b	Model 2 + educational level, occupational status, anti-hypertensive drugs
Model 2c	Model 2a + educational level, occupational status, anti-hypertensive drugs
Model 3	Model 2 + total energy intake, fruit, vegetables, cereals, red meat, processed meat, fish, margarine, sweet snacks, sugar-sweetened beverages, caffeinated coffee, tea, alcoholic beverages, dietary supplements
Model 4	Model 2 + Metabolite score
Secondary analyses	
Model a	C15:0, C17:0, C16:1n7t, and C14:0 fatty acids
Model b	Model a + model 0
Model c	Model b + total energy intake, fruit, vegetables, cereals, red meat, processed meat, fish, margarine, sweet snacks, sugar-sweetened beverages, caffeinated coffee, tea, alcoholic beverages, dietary supplements

Supplemental Table 3. Likelihood ratio (LR) tests and net reclassification improvement (NRI) for the models with or without the fatty acids[a] and the metabolite score in the fatty acid set of the Fenland study for internal validation (n=4,246) and EPIC-Norfolk study for external validation (n=592)

**Reference model** [b]	**Covariates added**	**Milk**			**Yogurt**			**Cheese**			**Butter**			**Total dairy products**		
		**LR**	**NRI**	p_{NRI}	**LR**	**NRI**	p_{NRI}	**LR**	**NRI**	p_{NRI}	**LR**	**NRI**	p_{NRI}	**LR**	**NRI**	p_{NRI}
**Fenland Study (internal validation)**																
Demographic, socio-economic lifestyle	Three fatty acids[a]	61	0.18	<0.001	27	0.14	0.01	63	0.34	<0.001	263	0.45	<0.001	199	0.58	<0.001
	Score with all 82 metabolites	219	0.45	<0.001	62	0.32	<0.001	90	0.48	<0.001	363	0.63	<0.001	331	0.88	<0.001
	Score with selected metabolites 2 SD criterion[c]	80	0.30	<0.001	22	0.26	<0.001	53	0.36	<0.001	212	0.46	<0.001	266	0.82	<0.001
	1 SD criterion[c]	99	0.32	<0.001	33	0.23	<0.001	60	0.41	<0.001	330	0.61	<0.001	306	0.83	<0.001
+ three fatty acids[a b]	Score with all metabolites	161	0.40	<0.001	43	0.24	<0.001	37	0.31	<0.001	122	0.40	<0.001	146	0.57	<0.001
	Score with selected metabolites 2 SD criterion[c]	47	0.22	<0.001	9	0.10	0.04	19	0.23	<0.001	54	0.27	<0.001	103	0.51	<0.001
	1 SD criterion[c]	51	0.25	<0.001	18	0.20	<0.001	22	0.27	<0.001	96	0.37	<0.001	132	0.51	<0.001
+ Score with metabolites (all 82)[b]	Three fatty acids[a]	3	0.02	0.61	8	0.01	0.09	10	0.10	0.08	22	0.15	<0.001	13	0.16	0.01
2 SD criterion [c]	Three fatty acids[a]	27	0.10	0.01	14	0.12	0.02	29	0.21	<0.001	106	0.27	<0.001	37	0.25	<0.001
1 SD criterion [c]	Three fatty acids[a]	14	0.11	0.01	11	0.06	0.13	24	0.19	<0.001	28	0.13	0.001	23	0.20	0.001
**EPIC-Norfolk (external validation)**																
Demographic, socio-economic lifestyle	Two fatty acids[a]	4	0.06	0.57	0	0.10	0.56	0	0.17	0.33	67	0.81	<0.001	16	0.82	<0.001
	Score with selected metabolites 2 SD criterion[c]	8	0.33	0.003	0	0.04	0.83	0	-0.03	1.15	1	0.27	0.007	8	0.51	0.03
+ two fatty acids	Score with selected metabolites, 2 SD criterion[c]	5	0.16	0.15	0	0.12	0.49	0	-0.07	1.33	0	0.05	0.60	1	0.23	0.31
+ Score with all metabolites [b]	Two fatty acids[a],	1	0.03	0.78	0	0.16	0.34	0	0.12	0.47	66	0.84	<0.001	9	0.60	0.01

a) Fatty acids: C15:0, C17:0, C16:1n7t in the Fenland Study and C15:0, C17:0 in the EPIC-Norfolk Study.
b) Socio-demographic and lifestyle factors: age, sex, test site, smoking status, physical activity, lipid lowering drugs, hormone-replacement therapy, and BMI
c) Selection of metabolites was based on the top metabolites defined as metabolites with absolute values of elastic net coefficients > mean+2×SD or >mean+SD
LR: likelihood ratio; NRI: net reclassification improvement

Supplemental Table 4. Areas under the curve of the logistic models[a] with or without the fatty acids C15:0, C17:0, C16:1n7t and the metabolite score (with selected metabolites [b]) for total and types of dairy products in the internal validation set of the Fenland study (n=4,246) and the external validation set of the EPIC-Norfolk diabetes case-cohort study (n=592).

**Internal validation set (Fenland study)**				**External validation set (EPIC-Norfolk)**			
**Milk**		Fatty acids		**Milk**		Fatty acids	
		No	Yes			No	Yes
Metabolite score	No	0.59	0.62	Metabolite score	No	0.61	0.62
	Yes	0.63	0.64		Yes	0.63	0.64
**Yogurt**		Fatty acids		**Yogurt**		Fatty acids	
		No	Yes			No	Yes
Metabolite score	No	0.67	0.69	Metabolite score	No	0.70	0.70
	Yes	0.68	0.69		Yes	0.70	0.70
**Cheese**		Fatty acids		**Cheese**		Fatty acids	
		No	Yes			No	Yes
Metabolite score	No	0.67	0.72	Metabolite score	No	0.70	0.71
	Yes	0.71	0.73		Yes	0.70	0.70
**Butter**		Fatty acids		**Butter**		Fatty acids	
		No	Yes			No	Yes
Metabolite score	No	0.59	0.70	Metabolite score	No	0.62	0.77
	Yes	0.69	0.72		Yes	0.63	0.77
**Total dairy products**		Fatty acids		**Total dairy products**		Fatty acids	
		No	Yes			No	Yes
Metabolite score	No	0.59	0.75	Metabolite score	No	0.59	0.76
	Yes	0.78	0.80		Yes	0.71	0.77

a) All models include age, sex, test site, smoking status, physical activity, lipid lowering drugs, hormone-replacement therapy and BMI
b) Metabolites approximately matched between the derivation (targeted) set and the external validation (untargeted) set. Selection of metabolites was based on the top metabolites defined as metabolites with absolute values of the elastic net coefficients >mean+2×SD

Supplemental Table 5. Hazard ratios of T2D per 1 SD of metabolite scores not significantly associated with dairy consumption after 16,360 person-years of follow-up (641 cases of T2D) in the incident diabetes case-cohort study within the EPIC Norfolk study

**Metabolite score for each dairy type Selected metabolites**[a]	**Model**	**HR**	**95% CI**	***p***
**Yogurt**	Socio-demographic + family history of T2D [b]	0.92	0.85, 1.00	0.053
	+Smoking, physical activity, drugs [c]	0.96	0.88, 1.04	0.292
	+ Diet [d]	1.04	0.95, 1.14	0.358
	+ BMI	1.05	0.96, 1.15	0.249
	+OCSFA	1.36	1.14, 1.61	<0.001
**Cheese**	Socio-demographic + family history of T2D [b]	0.81	0.74, 0.88	<0.001
	+Smoking, physical activity, drugs [c]	0.86	0.79, 0.93	<0.001
	+ Diet [d]	0.94	0.86, 1.03	0.184
	+ BMI	1.14	1.04, 1.26	0.006
	+OCSFA	1.35	1.15, 1.58	<0.001

a) Metabolite scores generated from the total sample of the incident diabetes case-cohort study (n=1,440) and standardized using the SD of the scores within the subcohort. Selected metabolites are defined as the metabolites with coefficients above mean+2×SD.
b) Models adjusted for age (continuous in years), sex, educational level (low, medium, high), socio-economic status (low, medium, high) and family history of T2D (yes, no)
c) Models additionally adjusted for smoking (never, former, current smoker), physical activity (inactive, moderately inactive, moderately active, active), lipid-lowering drugs (yes, no), anti-hypertensive drugs (yes, no), hormone-replacement therapy (yes, no, men)
d) Models additionally adjusted for total energy intake (kcal/day), dietary supplement use (yes, no), consumption (g/day) of fruit, vegetables, total cereals, red meat, processed meat, fish, margarine, sweet snacks, sugar-sweetened beverages, coffee, tea, alcoholic beverages
CI: confidence interval; EPIC: European Investigation into Cancer and Nutrition; HR: hazard ratio; OCSFA: odd-chain saturated fatty acids; T2D: type 2 diabetes

Supplemental Table 6. Classification ability of the metabolite scores for consumption of different dairy products: a secondary external validation analysis of the metabolite scores derived from the Fenland Study and tested in the EPIC-Norfolk Study, United Kingdom[a]

**Dairy outcome**	**Metabolite selection criterion**	**N metabolites** [b]	**N**	**Metabolite score**[c] **No AUC**	**Yes AUC**	**NRI**	p_{NRI} [d]
Milk	1SD	7	900	0.59	0.59	0.10	0.14
	All 82 metabolites	82	900	0.59	0.60	0.15	0.016
	2SD without SM-OH-C14:1	2	900	0.59	0.59	0.12	0.05
Yogurt	1SD	13	875	0.69	0.69	0.15	0.15
	All 82 metabolites	82	875	0.69	0.69	0.05	0.33
	2SD without SM-OH-C14:1	1	875	0.69	0.69	0.07	0.25
Cheese	1SD	7	475	0.65	0.67	0.26	0.02
	All 82 metabolites	82	475	0.65	0.66	0.08	0.24
	2SD without SM-OH-C14:1	3	475	0.65	0.66	0.08	0.22
Butter	1SD	11	1,193	0.61	0.61	0.08	0.27
	All 82 metabolites	82	1,193	0.61	0.62	0.15	0.015
	2SD without SM-OH-C14:1	5	1,193	0.61	0.62	0.14	0.019
Total dairy products	1SD	6	687	0.59	0.62	0.21	0.14
	All 82 metabolites	82	687	0.59	0.64	0.13	0.17
	2SD without SM-OH-C14:1	3	687	0.59	0.61	0.30	0.01

a) All the statistics were adjusted for socio-demographic and lifestyle factors including age, sex, test site, smoking status, physical activity, lipid lowering drugs, hormone-replacement therapy, and BMI
b) The set of metabolites used in the analysis to derive the metabolite scores in the Fenland Study using the different cut-off point of the mean+1SD for the absolute values of the elastic net coefficients
c) " No" refers to the model including covariates only; and " Yes" , the model including covariates and the metabolite score
d) *p* values for the metabolite score for the classification of consumers and non-consumers
AUC: area under the curve; EPIC: European Investigation into Cancer and Nutrition; NRI: net reclassification improvement

Supplemental Table 7. Associations of metabolite scores including selected top metabolites classifying each of total and types of dairy consumption with other dairy products and food groups in the EPIC Norfolk case-cohort study

	Metabolite score														
	Milk			Yogurt			Cheese			Butter			Total dairy products		
**Food group[a]**	**b**	**95% CI**	***P***	**b**	**95% CI**	***P***	**b**	**95% CI**	***P***	**b**	**95% CI**	***P***	**b**	**95% CI**	***P***
Milk	0.14	(0.03, 0.26)	0.02**	0.06	(-0.14, 0.26)	0.57	0.11	(-0.05, 0.28)	0.18	0.03	(-0.04, 0.11)	0.37	0.04	(-0.03, 0.10)	0.27
Yogurt	-0.10	(-0.20, -0.01)	0.03*	0.000	(-0.21, 0.22)	1.00	-0.01	(-0.14, 0.13)	0.94	0.004	(-0.07, 0.08)	0.91	-0.03	(-0.09, 0.04)	0.41
Cheese	0.06	(-0.03, 0.16)	0.21	0.14	(-0.04, 0.33)	0.14	0.08	(-0.13, 0.29)	0.48	0.04	(-0.04, 0.11)	0.36	-0.002	(-0.07, 0.06)	0.94
Butter	0.16	(0.06, 0.26)	0.002**	0.33	(0.12, 0.54)	0.002**	0.22	(0.08, 0.36)	0.002**	0.09	(0.005, 0.17)	0.04**	0.07	(-0.005, 0.14)	0.07
Total dairy products	0.21	(0.10, 0.31)	<0.001**	0.30	(0.09, 0.51)	0.01**	0.25	(0.10, 0.40)	0.001**	0.09	(0.01, 0.17)	0.02**	0.06	(0.000, 0.13)	0.05
Cereals	0.06	(-0.03, 0.16)	0.21	0.17	(-0.02, 0.36)	0.07	0.15	(0.01, 0.29)	0.04**	0.03	(-0.04, 0.11)	0.39	0.06	(0.001, 0.12)	0.05
Fruit	-0.01	(-0.11, 0.10)	0.92	0.24	(0.07, 0.42)	0.01**	-0.004	(-0.13, 0.13)	0.96	-0.005	(-0.08, 0.07)	0.90	0.02	(-0.04, 0.09)	0.44
Vegetables	0.004	(-0.09, 0.10)	0.94	0.30	(0.10, 0.50)	0.003**	0.01	(-0.14, 0.16)	0.90	0.08	(0.01, 0.16)	0.03**	0.06	(0.001, 0.11)	0.05
Potatoes	0.06	(-0.04, 0.17)	0.24	0.22	(-0.01, 0.44)	0.06	0.03	(-0.13, 0.19)	0.70	0.05	(-0.03, 0.12)	0.23	-0.02	(-0.08, 0.05)	0.65
Legumes	-0.03	(-0.13, 0.07)	0.57	0.21	(0.004, 0.42)	0.05	-0.15	(-0.30, 0.01)	0.07	0.02	(-0.06, 0.10)	0.60	-0.01	(-0.07, 0.05)	0.67
Nuts	0.01	(-0.11, 0.12)	0.93	0.20	(-0.04, 0.45)	0.11	-0.01	(-0.15, 0.13)	0.89	0.04	(-0.03, 0.11)	0.28	0.04	(-0.01, 0.10)	0.13
Poultry and eggs	-0.06	(-0.16, 0.04)	0.21	-0.15	(-0.35, 0.04)	0.12	-0.17	(-0.31, -0.02)	0.02*	-0.09	(-0.17, -0.02)	0.02*	-0.04	(-0.10, 0.01)	0.14
Red meat	0.05	(-0.06, 0.15)	0.37	-0.13	(-0.34, 0.08)	0.23	-0.08	(-0.20, 0.05)	0.24	0.03	(-0.05, 0.11)	0.47	0.03	(-0.03, 0.09)	0.35
Processed meat	0.01	(-0.08, 0.11)	0.77	-0.02	(-0.23, 0.20)	0.87	-0.04	(-0.18, 0.10)	0.59	-0.005	(-0.08, 0.07)	0.90	-0.05	(-0.10, 0.01)	0.12
Fish	-0.06	(-0.16, 0.04)	0.24	-0.15	(-0.35, 0.05)	0.14	0.000	(-0.14, 0.14)	1.00	-0.11	(-0.19, -0.04)	0.004*	-0.05	(-0.11, 0.02)	0.15
Margarines	-0.06	(-0.16, 0.04)	0.21	-0.16	(-0.37, 0.05)	0.14	0.07	(-0.07, 0.22)	0.33	-0.08	(-0.15, 0.004)	0.06	-0.02	(-0.08, 0.04)	0.49
Sugar-sweetened beverages	-0.06	(-0.16, 0.05)	0.27	-0.10	(-0.29, 0.09)	0.31	-0.08	(-0.22, 0.07)	0.30	-0.04	(-0.13, 0.04)	0.28	-0.08	(-0.15, -0.02)	0.01*
Artificially-sweetened beverages	-0.14	(-0.23, -0.04)	0.01*	-0.14	(-0.32, 0.05)	0.14	-0.11	(-0.26, 0.04)	0.16	-0.10	(-0.17, -0.02)	0.01*	-0.07	(-0.13, -0.02)	0.01*
Fruit juice	-0.15	(-0.25, -0.06)	0.001*	0.05	(-0.13, 0.24)	0.57	-0.003	(-0.17, 0.17)	0.97	-0.04	(-0.11, 0.04)	0.38	-0.03	(-0.08, 0.03)	0.39
Tea	0.09	(-0.01, 0.19)	0.07	0.23	(0.03, 0.42)	0.03	0.16	(0.01, 0.31)	0.03**	0.08	(0.00, 0.15)	0.05	0.07	(0.01, 0.12)	0.02**
Coffee	0.01	(-0.09, 0.11)	0.89	-0.15	(-0.35, 0.05)	0.14	0.02	(-0.13, 0.17)	0.82	0.01	(-0.07, 0.08)	0.87	-0.02	(-0.07, 0.04)	0.54
Alcoholic beverages	-0.24	(-0.32, -0.15)	<0.001*	-0.42	(-0.64, -0.20)	<0.001*	-0.28	(-0.40, -0.17)	<0.001*	-0.09	(-0.16, -0.01)	0.03*	-0.17	(-0.23, -0.12)	<0.001*

*b<0 and *P*<0.05

** b>0 and *P*<0.05

a) Food groups were standardised

CI: confidence interval; EPIC: European Investigation into Cancer and Nutrition

Supplemental Table 8. Hazard ratios of T2D per 1 SD of metabolite scores classifying dairy consumption in the incident diabetes case-cohort study within the EPIC Norfolk study[a]

Dairy type	Metabolites		Metabolites + phospholipid fatty acids	
	**1 SD**	**82 metabolites**	**2 SD**[b c]	**85 metabolites**[b]
Milk	0.85 (0.77, 0.93)	0.92 (0.84, 1.01)	0.82 (0.72, 0.94)	0.97 (0.86, 1.09)
Yogurt	1.47 (1.34, 1.61)	1.53 (1.41, 1.67)	1.35 (1.17, 1.55)	1.67 (1.48, 1.89)
Cheese	1.17 (1.06, 1.29)	1.29 (1.18, 1.42)	1.41 (1.22, 1.62)	1.61 (1.39, 1.85)
Butter	0.45 (0.41, 0.50)	0.44 (0.40, 0.49)	0.59 (0.51, 0.68)	0.35 (0.30, 0.41)
Total dairy products	0.70 (0.64, 0.77)	0.77 (0.70, 0.84)	0.76 (0.66, 0.87)	0.79 (0.70, 0.90)

[a] Based on analysis of 641 cases of T2D and 16,360 person-years of follow-up unless otherwise specified. Metabolite scores significantly associated with milk, butter, and total dairy consumption levels, but not yogurt and cheese consumption levels.

[b] HRs and 95% CIs were estimated per 1 SD difference in each metabolite score. Models adjusted for age (continuous in years), sex, educational level (low, medium, high), socio-economic status (low, medium, high) and family history of T2D(Yes, No), smoking (never, former, current smoker), physical activity (inactive, moderately inactive, moderately active, active), lipid-lowering drugs (yes, no), anti-hypertensive drugs (yes, no), hormone-replacement therapy (yes, no, men), total energy intake (kcal/day), dietary supplement use (yes, no), consumption (g/day) of fruit, vegetables, total cereals, red meat, processed meat, fish, margarine, sweet snacks, sugar-sweetened beverages, coffee, tea, alcoholic beverages, and BMI .

[c] Metabolite scores generated from the subset with OCSFA measurements of the incident diabetes case-cohort study (n=592) and standardized using the SD of the scores within the subcohort. Selected metabolites include top metabolites defined as the metabolites with absolute values of the elastic net coefficients >mean+2×SD.

CI: confidence interval; EPIC: European Investigation into Cancer and Nutrition; HR: hazard ratio; OCSFA: odd-chain saturated fatty acid; T2D: type 2 diabetes

Supplemental Figures

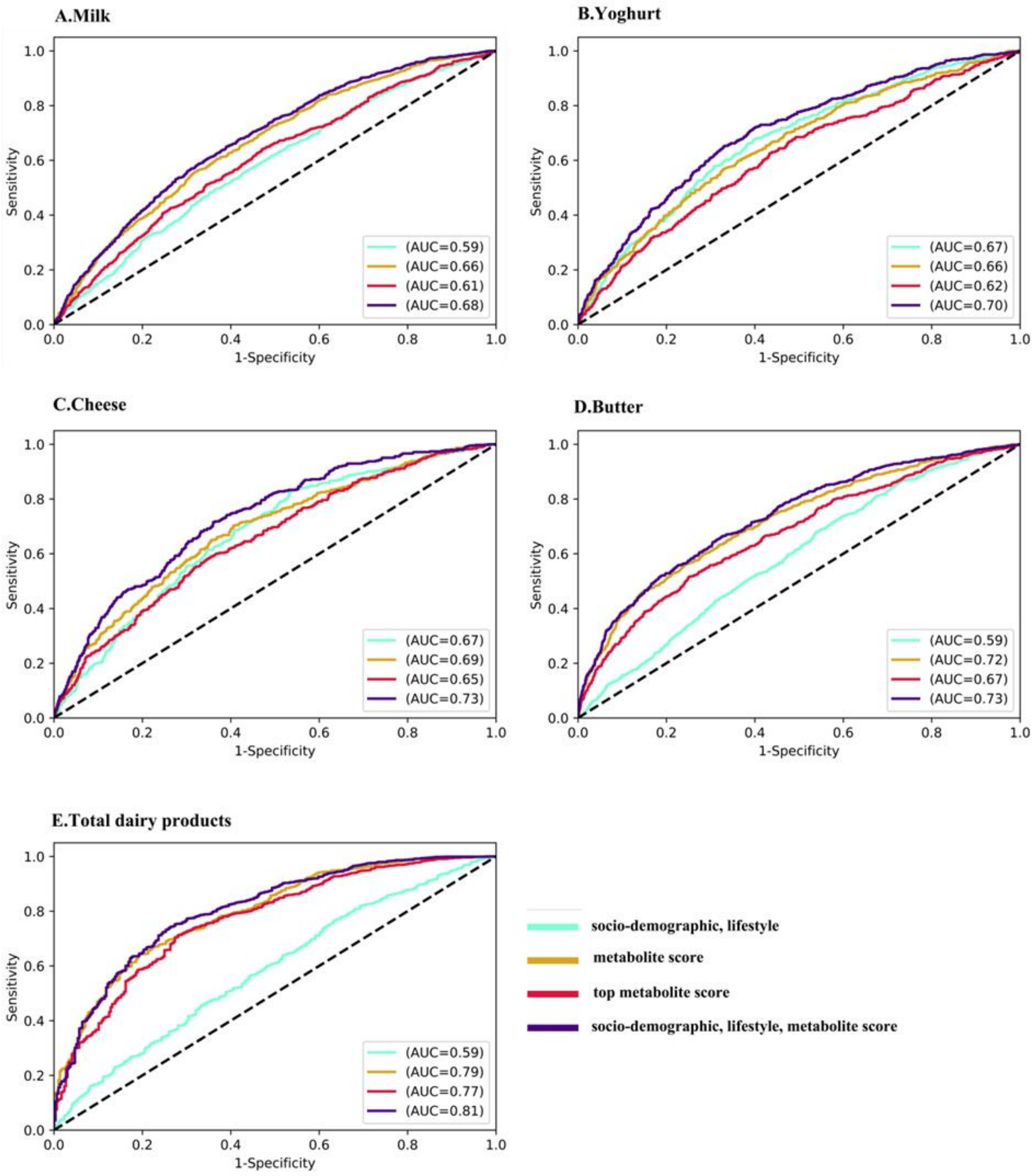

Supplemental Figure 1. Receiver operating characteristic (ROC) curves and corresponding AUCs for prediction models of metabolite scores reflecting (A) milk, (B) yogurt, (C) cheese, (D) butter and (E) total dairy consumption in the internal validation set (n=4,246). Socio-demographic, lifestyle model: age, sex, test site, smoking status, physical activity, lipid lowering drugs, hormone-replacement therapy and BMI; Top metabolite score: metabolites with absolute values of elastic net coefficients >mean+2×SD. AUC: area under the curve

Matched metabolite	Milk (high vs low consumers) †		Milk (energy density) ‡		Yogurt †		Cheese †		Butter †		Total dairy products (high vs low consumers) †		Total dairy products (energy density) ‡	
	Elastic net	Ordinary	Elastic net	Ordinary	Elastic net	Ordinary	Elastic net	Ordinary	Elastic net	Ordinary	Elastic net	Ordinary	Elastic net	Ordinary
Ala	-0.028	-143.2	-0.004	-0.01	-0.005	-64.39	-0.104	-222.51	0	-13.58	-0.022	-180.01	-0.009	-0.01
Arg	0.032	102.86	0.003	0	-0.006	-32.2	0	109.61	0.026	181.47	0.057	272.26	0.005	0.01
Asn	0	24.06	0	0	0.032	114.73	0.059	266.29	-0.044	-66.1	0	-123.06	0	0
Asp	0.05	185.38	0.007	0.01	-0.066	-234.11	0	-25.1	0	-102.6	0	19.5	0.002	0
Cit	0.113	203.81	0.015	0.02	0	-96.81	0	-98.78	0	-25.38	0	54.18	0.002	0.01
Gln	0.085	274.87	0.007	0.01	-0.021	-109.97	0.051	165.84	0	-62.22	0	-201.43	0	0.01
Glu	-0.024	-145.64	-0.004	-0.01	-0.063	-199.45	0.091	279.69	0.066	120.97	0	-20.85	0	0
Gly	-0.014	-136.6	-0.005	-0.01	-0.035	-51.06	0.1	285.89	-0.03	-23.52	0	-42.36	0	0
His	-0.118	-135.65	-0.02	-0.03	0.014	285.56	0	60.44	0	-66.11	0	-147.78	-0.015	-0.02
Ile	-0.238	-355.02	-0.021	-0.03	-0.109	-251.95	0	-186.26	0.01	119.87	-0.026	-179.13	-0.008	-0.01
Leu	0.084	200.62	0.006	0.01	0.003	29.3	0	39.63	0	-93.71	0	166.57	0	0
Lys	0.041	82.52	0.008	0.01	0.034	212.54	0	-36.29	-0.099	-301.79	0	88.64	0	0
Met	-0.014	-119.52	0	0	0.105	151.61	0	-38.63	0	-70.47	0	157.1	0	0
Orn	-0.106	-186.07	-0.019	-0.02	0	-67.52	0	-66	0.058	233.69	-0.083	-320.9	-0.002	-0.01
Phe	0.103	287.9	0.026	0.03	0.014	90.39	0	35.25	0	74.04	0.034	399.09	0.017	0.02
Pro	-0.071	-141.14	-0.004	-0.01	-0.014	-206.12	0	172.77	0	178.44	0	5.53	0	0
Ser	-0.153	-419.58	-0.014	-0.02	-0.024	-101.19	0.023	314.45	0	41.99	0	-66.77	-0.001	0
Thr	0.034	108.15	0.012	0.02	0	47.89	-0.01	-124.66	0	-106.31	0	-38.74	0.005	0.01
Trp	0.017	173.41	-0.005	-0.01	0.014	73.39	0.043	288.36	0	100.85	0	155.14	0	-0.01
Tyr	0.055	134.04	0.003	0.01	0	162.39	0	-96.59	0.036	239.26	0	112.4	0.006	0.01
Val	0	-149.04	0	0	-0.017	-142.48	0	0.25	0	37.57	0	-113.41	-0.005	-0.01
Ac-Orn	0.012	-6.87	0.003	0.01	0.024	107.43	0	24.69	0	-20.06	0	62.82	0.008	0.01
SDMA	-0.04	-35.1	-0.014	-0.02	0	50.99	0.022	154.12	0.001	166.84	0	-106.91	-0.005	-0.01
alpha-AAA	0.062	165.26	0.007	0.01	0	132.01	0	-58.21	0	116.28	0.019	297.41	0.008	0.01
creatinine	0.12	268.79	0.016	0.02	-0.026	-198.65	-0.105	-365.1	0	-122.46	0	-57.69	0	0
kynurenine	0.027	57.47	0.005	0.01	0.013	309.14	0.164	424.23	0	0.47	0.096	289.04	0.009	0.01
Met-SO	0.014	59.01	0	0	0.018	19.28	0	-6.2	0	-22.18	0	-50.1	0	0
trans-OH-Pro	-0.137	-449.84	-0.017	-0.02	-0.166	-513.99	-0.263	-629.3	0.023	118.23	-0.12	-454.74	-0.024	-0.03
sarcosine	-0.02	-56.74	-0.006	-0.01	-0.07	-183.02	0	-1.96	0	13.75	0	-82.83	-0.005	-0.01
serotonin	-0.078	-155.79	-0.003	-0.01	0.023	130.61	0	39.35	-0.017	-76.59	0	48.06	0	0
taurine	0.1	132.59	0.011	0.02	0.128	301.05	0	-146.51	-0.074	-184.24	0	-58.59	0.002	0.01
C0	0.097	213.61	0.025	0.03	0	4.83	-0.189	-466.51	-0.006	-151.6	0	41.21	0.003	0.01
C2	-0.044	18.77	-0.006	-0.01	0.094	187.21	0	58.19	0	71.75	0	-162.49	0	0.01
C3	0.091	220.01	0	0	0.018	79.02	0	-117.94	0	112.89	0	165.98	0	-0.01
C4	-0.037	-36.72	-0.001	0	0.01	1.24	0	80.88	0	-90.74	0	118.25	0	0
C4-OH	0.033	65.77	0.002	0.01	0	-73.7	0	-19.46	-0.049	-140.09	0	-83.43	0	0.01
C5:1	0.089	207.87	0.013	0.02	0.061	236.07	0.037	305.68	-0.057	-234.62	0	234.4	0.01	0.01
C5-M-DC	0	3.71	-0.001	0	0	55.74	-0.067	-275.6	0	-52.23	0	-85.59	-0.006	-0.01
C6	-0.048	-79.54	-0.016	-0.02	-0.006	-135.69	0	-58.32	0	81.35	-0.024	-29.21	0	-0.01
C8	-0.039	-69.3	0	-0.01	0	74.54	-0.027	-251.17	0	7.66	0	-24.39	-0.01	-0.03
C10	0	-99.19	0	0.02	0	-71.75	0	7.05	0	39.4	0	52.41	0	0.02
C12	0	-69.4	-0.007	-0.01	0	-69.71	0	95.97	0.05	20.61	0	-18.65	0	0
C14	0.017	102.44	0	0	0.039	114.73	0	91.44	0	-62.65	0	76.55	0	-0.01
C14:1	-0.182	-281.03	-0.016	-0.02	-0.118	-241.69	0	-64.15	0.037	84.54	0	-197.97	-0.014	-0.03
C16	0.094	114.09	0.015	0.02	-0.127	-222.31	0	76.38	0.022	186.72	0	179.98	0.011	0.02
C18	-0.105	-157.1	-0.004	-0.01	-0.117	-271.6	0	-114.57	0.051	212.19	0	66.97	-0.005	-0.01
C18:2	-0.075	-223.68	-0.006	-0.01	0.075	291.3	0	-57.8	-0.183	-430.61	-0.051	-329.48	-0.018	-0.02
LPC a C14:0	0	56.28	0	0	0.004	195.57	0.071	248.36	0.233	396.58	0.172	405.69	0.012	0.02
LPC a C16:1	0.097	97.78	0.019	0.02	0.086	261.99	0.032	115.53	0	-93.16	0	105.06	0.005	0

LPC a C17:0	0.166	345.71	0.024	0.03	0.13	274.54	0.086	458.1	0.267	631.87	0.4	742.72	0.071	0.08
LPC a C18:0	-0.159	-233.95	-0.034	-0.07	0	-164.56	-0.215	-340.66	-0.141	-224.13	-0.424	-354.05	-0.072	-0.1
LPC a C18:2	-0.069	-138.4	-0.02	-0.01	0	10.94	0	-49.5	0	-110.81	-0.077	-325.02	-0.01	-0.01
LPC a C20:4	-0.122	-116.93	-0.014	-0.03	-0.076	-184.36	0	-107.99	0	-0.76	0	-232.63	0	-0.02
LPC a C16:0	-0.077	-205.26	0	0.02	0	-4.86	0	-151.22	-0.156	-264.12	0	-209.63	0	0.01
LPC a C18:1	-0.104	-177.67	-0.013	-0.03	0	-109.48	0	-10.17	0	30.14	0	-249.64	0	0.01
LPC a C20:3	0.324	435.63	0.041	0.06	0	127.2	0	13.56	-0.093	-178.95	0	146.22	0.008	0.02
PC aa C30:0	-0.043	22.17	-0.008	-0.01	0.086	153.29	0.074	297.54	0	142.76	0	301.5	0	0
PC aa C32:0	0.073	79.44	0.016	0.02	0.011	50.06	0	-51.25	-0.223	-530.32	0	-42.55	0	0
PC aa C32:1	0.023	91.95	0	0	0	-3.81	0	77.89	0	101.32	0.098	286.85	0.015	0.02
PC aa C32:2	-0.03	-36.13	-0.014	-0.02	0	54.99	0	109.41	0	40.36	0	107	-0.006	-0.02
PC aa C34:1	0	-101.4	0	0.01	0	-115.2	0	-52.8	0	63.03	0	-76.25	0	0
PC aa C34:2	0.037	-100.01	0	0	-0.053	-163.65	0	-3.56	-0.046	-162.96	0	-241.64	-0.011	-0.01
PC aa C36:1	-0.097	-154.72	-0.008	-0.01	-0.103	-329.11	0	-227.96	0.031	123.28	0	-162.61	0	-0.01
PC aa C36:2	0	-123.58	0	0.01	-0.118	-310.97	0	-249.56	0	-127.58	-0.014	-442.53	0	0.01
PC aa C36:3	0	73.23	0	-0.01	0.058	40.6	0	-3.92	-0.002	-105.87	0	-75.23	0	-0.01
PC aa C36:4	-0.188	-289.72	-0.009	-0.02	0	-99.45	0	-24.1	0	-51.02	0	-211.34	0	0
PC aa C36:5	-0.073	-106.71	-0.016	-0.03	0.047	166.75	0	29.33	-0.015	-203.1	0	-182.15	-0.004	-0.02
PC aa C38:4	0.068	28.58	0	0.01	-0.043	-242.47	-0.005	-179.69	0	-104.86	0	-224.23	0	0.01
PC aa C38:5	0.139	107.77	0.025	0.04	0	26.64	0	-69.76	-0.127	-183.34	0	-114.37	0	0.03
PC ae C34:1	0.042	151.6	0.008	0.01	0	25.27	0	166.8	0.283	358.06	0	373.82	0.016	0.01
PC ae C34:2	-0.033	-18.57	-0.005	-0.01	0	-76.87	0	-110.28	0	217.65	0	103.34	-0.007	-0.01
PC ae C36:1	0.007	107.66	0	-0.01	0	16.17	0	79.22	0.113	300.63	0	394.36	0	0
PC ae C36:2	0	115.59	0	0.01	0	93.88	0.09	428.37	0.138	399.75	0	523.12	0.014	0.03
SM C16:0	-0.157	-162.65	-0.026	-0.03	-0.108	-263.87	-0.152	-160.72	0	-120.39	-0.217	-379.81	-0.038	-0.04
SM C16:1	-0.279	-339.49	-0.058	-0.06	0	57.67	0	-188.62	-0.126	-195.51	-0.462	-414.27	-0.058	-0.06
SM C18:0	0.144	245.66	0.008	0.02	0	13.04	-0.028	-39.94	0	24.3	0	87.16	0	0.01
SM C18:1	-0.163	-256.47	-0.009	-0.02	0	173.66	-0.16	-191.42	0	82.3	-0.074	-131.61	0	-0.01
SM C24:1	-0.057	-236.52	-0.002	0	-0.062	-191.34	0	-106.07	-0.098	-376.26	-0.273	-526.66	-0.013	-0.01
SM-OH C14:1	0.587	817.2	0.104	0.11	0.271	494.28	0.475	742.89	0.407	750.77	1.185	1,174.93	0.141	0.15
SM-OH C22:1	-0.117	-172.57	-0.019	-0.02	0	-24.45	0	87.84	0	85.34	0	-32.75	-0.008	-0.02
SM-OH C22:2	0.082	243.78	0.01	0.01	0.016	138.6	0	184.16	0	72.31	0	243.68	0.002	0.01
hexose	0.059	125.31	0	0	-0.012	-112.99	0.034	189.88	0	-179.92	0	-164.59	-0.002	-0.01
age	-0.015	-4.34	0.001	0	0.026	37.51	-0.026	-68.87	-0.004	-32.53	0	11.87	0.001	0
sex	-0.31	-213.74	0	-0.07	0	213.44	0	268.87	0	-47.2	0	-67.03	0	0.03
test site	0.268	609.15	0.042	0.04	-0.023	-154.4	-0.094	-313.19	-0.262	-519.75	0	47.97	0	0
smoking status	-0.027	-99.73	-0.004	-0.01	-0.299	-792.13	0	-9.69	0.1	186.15	0	-133.9	0	0
physical activity	0.012	-14.39	0	0	0.012	-3.68	0	0.28	0	-2.88	0.014	7.42	0	0
lipid-lowering	-0.129	-68.16	0	-0.02	-0.006	-89.04	0	41.1	0	-44.65	0	-9.88	0	-0.03
HRT	0	207.05	-0.003	-0.04	-0.337	-633.63	-0.204	-432.65	0	104.98	0	104.3	-0.028	-0.02
BMI	0	6.67	0.001	0	0.011	30.79	0.014	142.3	0	-27.84	0.053	55.05	0.004	0
intercept	-0.036	-22.92	0.753	0.94	-1.594	-44.01	0.396	18.19	-0.689	4.09	0.262	-7.31	1.229	1.17

Supplemental Figure 2. Strength of associations of metabolites, socio-demographic and lifestyle covariates with consumption levels of total and types of dairy products in the derivation set of the Fenland study with the 82 metabolites matched with those in the diabetes case-cohort of the EPIC Norfolk study. Colors show the relative strength of associations within each column. Intensity of red color indicates strength of positive associations and intensity of blue color indicates strength of negative associations. † Strengths of associations are expressed as the beta coefficients of elastic net penalized logistic regression and ordinary logistic regression. ‡ Strengths of associations are expressed as the beta coefficients of elastic net penalized linear regression and ordinary linear regression. EPIC: European Prospective Investigation into Cancer and Nutrition

Matched metabolite	Milk (high vs low consumers) †		Milk (energy density) ‡		Yogurt †		Cheese †		Butter †		Total dairy products (high vs low consumers) †		Total dairy products (energy density) ‡	
	Elastic net	Ordinary	Elastic net	Ordinary	Elastic net	Ordinary	Elastic net	Ordinary	Elastic net	Ordinary	Elastic net	Ordinary	Elastic net	Ordinary
C15:0	2.426	443.51	0.005	0	39.101	328.3	27.003	959.96	14.813	744.32	52.447	1,027.88	0.045	0.04
C17:0	2.238	82.42	0	0	0	297.79	0	202.87	8.25	88.38	27.18	284.11	0	0
Ala	-0.617	-271.58	-0.013	-0.01	8.536	55.32	0	-27.93	0.88	176.97	0	-204.17	-0.003	0
Arg	0.904	-93.5	0	0	-38.535	-151.18	-1.092	-193.71	0	90.31	2.831	-80.4	0	0
Asn	-7.052	-164.49	-0.007	-0.01	7.624	272.71	0	237.78	-3.035	-257.34	1.847	142.18	-0.001	0
Asp	0	210.72	0	0	-26.629	-115.68	0	-32.84	2.122	254.94	14.076	327.84	0.003	0.01
Cit	7.53	283.94	0.007	0.01	-4.001	-53.67	5.417	224.9	0	-120.62	9.325	168.49	0	0
Gln	0	126.66	0	0	-55.038	-60.89	-6.335	-241	0	-2.56	0.474	-94.85	-0.001	0
Glu	10.809	9.35	-0.005	-0.01	-28.274	-257.36	-0.06	-80.66	3.587	250.16	4.781	90.6	-0.002	-0.01
Gly	-5.911	10.57	0.006	0.01	12.68	215.73	3.313	229.91	0	-290.17	-6.682	-173.76	0	0
His	0	141	-0.001	-0.01	-21.831	128.96	0	340.01	-0.293	-38.74	0	-52.43	0	0
Ile	2.085	-126.55	0	0.02	-68.856	-267.95	-7.384	-112.66	0	91.12	-2.49	-111.96	0	0.02
Leu	5.126	-14.68	0	0	-25.897	-35.66	-11.717	-148.19	0	-10.84	-2.115	-137.03	-0.005	-0.01
Lys	7.861	189.9	0.012	0.02	56.085	215.42	-8.866	-302.81	0	-76.33	16.997	84.85	0	0.01
Met	0	-70.89	0.001	0	-49.68	-212.27	-2.595	5.32	-1.179	-129.02	8.885	40.51	0	-0.01
Orn	0	-19.9	-0.008	-0.02	-49.666	-172.79	-5.844	-141.81	1.165	112.9	5.678	48.15	0	0
Phe	11.32	220.83	0.008	0.01	-17.853	-95.61	-1.129	6.1	0	22.11	7.386	62.79	0.002	0.01
Pro	-1.7	-291.83	-0.006	-0.01	-13.774	-76.99	0	66.07	0	83.37	9.793	33.94	0	0
Ser	-12.369	-213.52	-0.003	-0.01	-37.964	-124.87	0	-58.36	0	-112.42	-16.972	-264	-0.011	-0.01
Thr	-3.278	-137.72	-0.003	-0.01	23.171	141.8	0	94.08	0	53.26	-11.354	-446.45	-0.004	-0.01
Trp	8.237	186.32	0.004	0.01	-36.231	8.64	0	100.96	0	-166.86	12.103	135.66	0	0
Tyr	8.324	374.59	0.01	0.02	37.632	251.81	-0.011	147.44	0	251.67	13.794	235.26	0.014	0.02
Val	0	-239.25	-0.009	-0.03	-37.005	-70.1	-6.874	2.3	0	-107.55	-5.794	-269.06	0	-0.01
Ac-Orn	0	-146.65	0	0	23.472	178.76	0	117.93	0	35.78	-8.708	-152.85	0.001	0
SDMA	0	15.09	-0.008	-0.01	0	149.7	0	72.05	0	206.07	4.369	-40.05	0	0
alpha-AAA	8.487	8.85	0.003	0.01	16.297	151.22	-4.674	-26.07	0	-44.68	14.774	75.92	0	0
creatinine	8.939	313.4	0.019	0.03	-5.752	-176.43	-8.927	-451.03	0	106.05	10.535	77.39	0.006	0.01
kynurenine	6.529	245.02	0	0	-35.674	-81.76	0	-7.35	1.221	206.47	3.848	137.66	0	0
Met-SO	0	-65.11	0.002	0.01	-25.577	-176.48	0	253.27	0	-168.55	4.817	-15.23	0	0
trans-OH-Pro	-1.133	-207.05	-0.009	-0.01	48.748	-80.29	-16.318	-831.72	0	-6	-10.204	-488.35	-0.019	-0.02
sarcosine	0	-25.74	0	0	26.511	111.84	0	105.1	0	-16.27	3.333	97.76	0.002	0
serotonin	0	20.65	0.004	0.01	31.621	420.96	0	298.01	0	-3.98	13.114	302.18	0.006	0.01
taurine	0	142.34	0.012	0.02	68.016	217.02	0	-267.7	0	-197.41	18.555	181.21	0.006	0.01
C0	9.259	58.64	0.007	0.02	28.889	110.72	-15.011	-348.05	0	29.73	17.078	2.41	0.001	0.02
C2	0	-189.34	-0.006	-0.01	13.543	458.49	-4.973	177.29	0	-19.17	-5.961	9.65	0	0
C3	7.567	78.54	0.002	0	0	24.58	-16.556	-322.92	0	-64.31	10.169	58.14	-0.005	-0.02
C4	0	13.8	0	0	67.413	333.06	0	292.27	0	-40.73	6.762	38.67	0	0
C4-OH	9.146	115.86	0	0	7.237	152.11	-2.029	-174.51	0	-57.76	15.609	-9.27	-0.001	-0.01
C5:1	3.562	111.5	0.01	0.01	35.48	159.06	0	63.94	0	-233.92	12.801	343.3	0.011	0.02
C5-M-DC	5.622	128.98	0	0	44.117	169.61	-0.321	-74.59	0	-4.95	11.674	-45.82	0	0
C6	0	8.41	0	0.01	-27.999	-68.32	-4.719	-136.29	0	-31.1	3.585	87.19	0	0
C8	-4.484	-21.29	-0.003	-0.01	-19.019	21.06	-1.798	59.12	0	90.26	2.681	-12.88	0	0.01
C10	-4.579	-29.92	0	0	-44.263	-112.19	0	73.52	0	46.57	4.837	51.98	0	-0.02
C12	-2.827	-101.84	0	0	-33.842	-118.58	0	123.16	0	71.43	2.625	48.1	0	0.02
C14	0	13.21	0	0	-10.654	-18.36	0	-17.05	0	91.61	3.112	97.22	0	0
C14:1	-2.051	-185.48	-0.012	-0.02	-39.666	-40.92	-5.658	-77.05	0	-121.89	-9.359	-236.97	-0.007	-0.02
C16	1.31	22.23	0	0.01	-2.472	-37.39	-3.639	-75.08	0	9.31	12.355	220.95	0	0.01
C18	0	-138.95	-0.003	-0.01	-17.061	-345.41	0	-104.47	0	276.22	0	-127.19	-0.005	-0.01

C18:2	0	-104.27	0.004	0.01	-74.454	-71.77	-12.017	-232.93	0	-261.75	-18.628	-356.57	-0.002	0
LPC a C14:0	0	-289.23	-0.021	-0.04	91.298	321.66	19.561	379.97	6.985	216.92	35.086	343.8	0	0
LPC a C16:1	11.522	398.4	0.054	0.08	67.546	205.32	7.696	125.54	0	-215.48	25.97	126.21	0.041	0.05
LPC a C17:0	1.123	221.25	0.004	0.03	0	182.67	10.449	264.08	7.964	459.26	34.98	515.65	0.023	0.04
LPC a C18:0	0	-105.15	-0.017	-0.04	-43.24	-16.45	-0.474	-122.54	0	-124.5	-0.677	-185.04	-0.034	-0.02
LPC a C18:2	-9.679	-301.5	-0.037	-0.05	-64.525	-51.14	0	175.27	0	-14.82	-12.448	-154.69	-0.006	-0.01
LPC a C20:4	0	69.18	0	-0.01	-27.828	-161.1	-3.009	-138.5	-4.392	-138.07	-1.832	12.11	0	-0.02
LPC a C16:0	0	-150.95	0	-0.02	-12.311	36.48	0	-48.71	0	-151.12	1.771	-133.88	-0.009	-0.05
LPC a C18:1	0	-143.25	0	0	-31.851	-121.77	4.989	160.81	0	66.66	5.373	69.31	0	0.03
LPC a C20:3	4.248	411.74	0.018	0.03	22.359	115.28	0	-10.04	0	-5.89	8.77	232.82	0	0
PC aa C30:0	0	-183.54	0	0.02	64.025	198.7	23.537	651.29	5.854	160.56	31.759	293.38	0.013	0.03
PC aa C32:0	0	-117.38	-0.007	-0.02	18.171	34.1	0	-3.26	0	-333.21	3.43	-200.05	-0.008	-0.02
PC aa C32:1	8.201	266.23	0	-0.02	65.907	9.08	12.777	310.47	0.604	27.64	22.819	143.17	0.007	0.01
PC aa C32:2	0	-191.16	-0.009	-0.01	11.823	-13.64	12.394	181.43	4.445	-11.19	10.96	2.2	-0.028	-0.04
PC aa C34:1	0.984	7.55	0	0.01	7.826	-179.86	4.36	79.57	0	76.59	10.231	37.71	0.018	0.04
PC aa C34:2	-7.415	-297.79	-0.011	-0.01	-47.846	-198.35	0	-1.19	0	-27.64	-16.034	-410.01	-0.005	-0.01
PC aa C36:1	0	-167.04	-0.002	-0.01	0	-319.8	3.722	-49.37	0	23.87	7.482	-130.47	-0.007	-0.03
PC aa C36:2	-2.609	-119.78	0	0.02	-46.457	-120.78	0	-165	0.093	0.71	-15.68	-343.53	0	0.01
PC aa C36:3	0	173.69	0	-0.01	6.619	106.97	0	-173.27	0	-65.54	0	-82.7	0	0
PC aa C36:4	0	57.85	0	0.01	22.001	79.68	-1	-189.69	0	-150.5	-5.703	-219.72	0.001	0.02
PC aa C36:5	0	-284.48	-0.003	-0.02	14.353	203.45	0	15.08	0.879	-69.22	18.351	-91.58	0	-0.01
PC aa C38:4	3.447	208.45	0.009	0.01	-3.974	-57.73	-9.643	-327.06	-1.847	-271.35	-11.614	-255.35	0	0
PC aa C38:5	5.81	159.64	0.001	0.03	-13.973	-45.42	0	-101.68	0	-149.43	4.757	-78.47	0	0.01
PC ae C34:1	0	308.35	0.003	0.01	-0.637	-160.5	11.837	71.32	7.038	416.83	30.492	399.8	0	0
PC ae C34:2	-6.987	-124.34	0	0	-22.435	-220.53	0.49	-320.65	4.43	148.24	2.709	-24.18	0	-0.01
PC ae C36:1	1.979	75.66	0	0	34.286	7.89	9.927	97.74	7.978	404.56	24.162	183.97	0	0
PC ae C36:2	0	134.96	0	-0.02	-14.466	-14.92	12.718	274.04	9.662	438.12	21.526	334.66	0.006	0.01
SM C16:0	0	-50.59	0	0.01	-30.344	-151.92	0	-95.85	0	-308.13	0	-231.74	-0.028	-0.02
SM C16:1	0	-382.36	-0.077	-0.1	11.284	55.32	0	-150.8	0	-392.82	-1.306	-415.52	-0.091	-0.12
SM C18:0	0.751	41.74	-0.002	-0.03	55.802	24.84	0	-301.4	2.829	134.23	17.456	76.58	0	-0.02
SM C18:1	0	-180.36	0	0.02	66.717	199.66	0	-28.9	1.641	-1.18	12.017	-138.53	0.002	0.03
SM C24:1	-1.327	-202.58	0	0	-62.599	-225.91	4.862	79.56	0	4.05	0	-161.27	-0.003	0
SM-OH C14:1	9.48	837.69	0.108	0.13	97.587	453.08	13.497	459.71	10.103	615.33	57.437	1,018.34	0.162	0.18
SM-OH C22:1	0	-140.43	-0.023	-0.03	-0.888	-279.24	0.695	-1.23	1.714	135.26	8.257	56.4	-0.008	-0.02
SM-OH C22:2	0	66.91	0.006	0.01	62.936	257.57	4.265	12.26	2.936	-3.2	22.857	313.29	0.003	0.01
hexose	0	-240.45	-0.008	-0.01	2.341	136.51	-4.611	-136.43	0	-109.16	0.786	-132.01	-0.008	-0.01
age	0.026	40.02	0.001	0	0	9.26	-4,935.93	-47.13	0	-35.11	0.007	54.46	0.001	0
sex	0	-313.64	0	0.07	8.776	357.84	41,210.73	355.42	0	-183.81	0	-105.01	0	0.21
test site	0.193	493.9	0.027	0.03	0.602	-149.59	-66,489.21	-667.3	-0.194	-425.19	0	-122.97	-0.006	-0.01
smoking status	0.031	-5.39	0.005	0.01	-2.66	-676.41	11,028.38	25.13	0.151	253.24	0	29.99	0.012	0.02
physical activity	0.009	57.5	0	0	0	-40.73	1,604.98	-60.44	0	48.69	0.008	62.2	0	0
lipid-lowering	-0.087	-94.31	0	-0.05	-0.232	27.96	14,992.73	14.84	-0.044	-118.14	0	-33.69	0	-0.04
HRT	0.104	186.5	0	0.03	-0.372	-837.98	-78,063.18	-691.88	0	58.15	0	2.39	-0.034	0.06
BMI	0.051	-19.89	0.002	0	0	-5.4	5,585.89	71.99	0.019	31.24	0.055	123.08	0.005	0.01
intercept	-3.675	-84.54	0.727	0.54	-24.322	-25.94	350.666	1.02	-1.612	-55.64	-0.665	-42.24	1.182	0.69

Supplemental Figure 3. Strength of associations of metabolites, socio-demographic and lifestyle covariates with consumption levels of total and types of dairy products in the derivation set of the Fenland study with the 82 metabolites matched with those in the diabetes case-cohort of the EPIC Norfolk study and the OCSFAs. Colors show the relative strength of associations within each column. Intensity of red color indicates strength of positive associations and intensity of blue color indicates strength of negative associations. † Strengths of associations are expressed as the beta coefficients of elastic net penalized logistic regression and ordinary logistic regression. ‡ Strengths of associations are expressed as the beta coefficients of elastic net penalized linear regression and ordinary linear regression. EPIC: European Investigation into Cancer and Nutrition; OCSFA: odd-chain saturated fatty acid

Metabolite abbreviation (targeted)	Milk (high vs low consumers) †		Milk (energy density) ‡		Yogurt †		Cheese †		Butter †		Total dairy products (high vs low consumers) †		Total dairy products (energy density) ‡	
	**Elastic net**	**Ordinary**	**Elastic net**	**Ordinary**	**Elastic net**	**Ordinary**	**Elastic net**	**Ordinary**	**Elastic net**	**Ordinary**	**Elastic net**	**Ordinary**	**Elastic net**	**Ordinary**
Ala	-0.02	-114.67	-0.003	-0.01	-0.149	-13.48	0	-272.27	0	-29.82	0	-254.37	-0.008	-0.01
Arg	0.029	138.86	0.002	0.01	0	-134.95	2.21	133.37	0.036	163.45	0.066	293.77	0.004	0.01
Asn	0	-68.2	0	0	0.172	101.3	0	265.65	-0.034	-114.78	0	-95.21	0	0
Asp	0.024	79.94	0.003	0.01	-0.527	-143.26	0	-143.11	0	-167.32	0	6.22	0	0
Cit	0.105	217.88	0.013	0.02	-0.155	-30.7	0	-120.49	0	-55.15	0	185.83	0	0.01
Gln	0.054	120.65	0.001	0.01	-0.319	-107.7	1.723	257.6	0	-88.47	0	-120.86	0	0
Glu	0	-146.37	-0.002	-0.01	-0.335	-174.86	2.239	238.95	0.058	110.06	0	-110.02	0	0
Gly	-0.008	-162.41	-0.002	-0.01	0	-41.12	0	232.25	-0.019	-99.37	0	-3.68	0	0
His	-0.085	-116.73	-0.014	-0.03	0.403	162.94	0	112.92	0	125.97	0	-112.58	-0.011	-0.02
Ile	-0.203	-305.32	-0.016	-0.03	-0.482	-287.47	0	-199.09	0.015	164.27	0	-192.46	-0.01	-0.01
Leu	0.059	119.68	0.001	0.01	0	54.41	0	19.44	0	-92.75	0	231.49	0	0
Lys	0.042	53	0.007	0.01	0.267	108.23	0	-6.18	-0.052	-231.91	0	134.56	0	0
Met	0	-50.34	0	0	0.339	16.11	0	-58.39	0	4.08	0	165.47	0	0
Orn	-0.1	-212.74	-0.018	-0.02	0	-56.12	0	-136.1	0.036	208.39	-0.067	-319.86	-0.003	-0.01
Phe	0	8.1	-0.002	0	0	-14.93	0	-45.55	0	-68.67	0	-111.79	0	0
Pro	0.099	288.2	0.023	0.03	0	61.11	0	33.73	0	33.32	0	301.76	0.014	0.02
Ser	-0.061	-239.99	-0.003	-0.01	-0.205	-105.31	0	116.56	0	87.98	0	58.99	0	0
Thr	-0.145	-337.07	-0.011	-0.01	0	-134.35	1.112	390.4	0	39.28	0	-97.67	0	0
Trp	0.019	126.05	0.01	0.02	0	34.49	0	-80.47	-0.041	-22.42	0	-6.99	0.004	0.01
Tyr	0.027	158.53	0	-0.01	0	67.62	3.335	324.21	0	90.69	0	204.32	0	-0.01
Val	0.033	109.37	0	0.01	0.221	202.22	0	-32.32	0.039	153.94	0	103.31	0.003	0.01
PEA	0	-112.66	0	0.01	-0.332	-97.37	0	15.24	0	81.43	0	-93.43	0	-0.01
AcOrn	0	-13.05	0	0.01	0.131	60.06	0	-3.61	0	9.25	0	84.73	0.007	0.01
SDMA	-0.024	-52.08	-0.01	-0.02	0	107.97	0.898	265.52	0	138.2	0	-118.35	-0.002	-0.01
alpha-AAA	0.068	211.43	0.007	0.01	0	112.38	0	-67.36	0	93.25	0	309.63	0.008	0.01
Creatinine	0.109	370.36	0.012	0.02	0	-186.04	-1.918	-269.78	0	-114.55	0	-49.83	0	0
Kynurenine	0	41.5	0.001	0	0.331	264.98	0	411.41	0	-32.44	0	223.1	0.006	0.01
Met-SO	0	-17.54	0	0	0	49.82	0	-39.72	0	-61.01	0	-106.67	0	0
c4OHPro	-0.01	-159.83	0	0.01	-0.836	-218.79	-1.732	-340.11	0	15.96	-0.049	-320.43	-0.004	0
t4OHPro	-0.108	-192.47	-0.012	-0.02	-0.311	-190.87	-0.693	-368.29	0.015	109.23	-0.066	-349.23	-0.015	-0.02
Sarcosine	-0.006	-64.45	-0.004	-0.01	-0.309	-178.76	0	-119.2	0	-35.5	0	-159.71	-0.003	-0.01
Serotonin	-0.066	-110.12	-0.001	-0.01	0.158	180.74	0	118.91	-0.019	-20.34	0	100.98	0	0
Spermidine	0.028	24.93	0.009	0.01	0	24.05	-0.716	-214.37	-0.011	-81.66	0	15.86	0.003	0.01
Taurine	0.08	158.92	0.006	0.01	0.709	274.6	0	-34.42	-0.069	-179.55	0	-47.46	0.001	0.01
C0	0.06	203.5	0.019	0.03	0	61.14	-3.568	-400.74	0	-186.54	0	40.21	0.001	0.01
C2	0	-52.77	-0.001	-0.02	0.458	158.58	0	204.66	0	60.92	0	-170.34	0	0
C3	0.082	240.65	0	0	0.031	163.59	-0.386	-124.27	0	78.88	0	201.87	0	0
C31	0	-58.48	0	0	0	49.34	0	-112.07	-0.048	-126.67	0	-16.2	0	0
C3OH	0	-26.43	0	0	0	-25.08	0	-44.61	0	-15.02	0	-83.8	0	0.01
C4	0	-22.51	0	0	0.272	65.43	0	176.32	0	-66.39	0	124.85	0.001	0
C41	-0.051	-106.8	-0.003	-0.01	0	36.09	0	-123.3	0	31.89	0	169.08	0	-0.01
C3DCC4OH	0.024	74.47	0	0.01	0	-103.91	-0.53	-69.56	-0.049	-58.94	0	-18.04	0	0.01
C5	0	-16.34	0	-0.01	-0.388	-200.04	0	-51.19	0	101.78	0	-13.52	0	-0.01
C51	0.07	101.55	0.012	0.02	0.271	152.45	3.213	337.7	-0.017	-149.95	0	175.58	0.012	0.02
C51DC	0	34.71	-0.003	-0.01	-0.058	-51.45	0	86.09	0	3.68	0	-174.87	-0.004	-0.01
C5DCC6OH	0	-31.55	0	0	0	8.75	0	-15.95	0	38.72	0	107.51	0	0.01
C5MDC	0	-28.31	0	0	0	94.22	-2.423	-252.75	0	-11.63	0	1.95	-0.003	-0.01
C3DCMC5OH	0	-25.76	-0.003	-0.01	0	148.11	0.079	5.74	-0.011	-127.64	-0.075	-365.39	-0.002	-0.01
C6C41DC	-0.053	-183.24	-0.012	-0.02	0	-71.93	0	-97.67	0	51.85	0	-18.28	0	0

C61	0.007	177.75	0	0	-0.179	-72.79	-0.122	38.03	0	5.18	0	47.5	-0.001	-0.01
C7DC	0	103.01	0	0	0	70.15	0	-160.22	0	100.09	0	6.22	0	0
C8	-0.021	-76.44	0	0	0	19.91	0	-180.56	0	3.4	0	-27.74	-0.005	-0.02
C9	0	-55.86	0	-0.01	-0.602	-267.99	0	106.37	0.236	449.27	0	117.89	0	0
C10	0	-121.25	0	0.01	-0.015	-129.11	1.626	95.94	0	86.79	0	63.64	0	0.02
C101	0	7.53	0	0	-0.034	-76.31	-1.572	-329.79	0	-61.52	0	-80.28	0	0
C102	0	15.4	0	0	0.492	281.21	-1.112	-219.42	-0.13	-307.26	0	-30.57	-0.001	0
C12	0	-78.09	-0.005	-0.02	0	-103.47	0	174.78	0	65.87	0	-27.41	0	0
C12DC	0	-27.04	0	0	0	51.68	0	62.92	-0.024	-84.71	0	-50.05	0	-0.01
C121	0.055	101.94	0	0.02	0	-13.75	-0.556	-69.49	0	94.57	0	166.09	0	0.02
C14	0	-16.29	0	-0.01	0.353	137.69	0	63.98	0	-28.28	0	48.66	0	-0.01
C141	-0.208	-321.08	-0.017	-0.04	-0.561	-212.4	-0.623	18.97	0.077	138.3	0	-202.16	-0.012	-0.04
C141OH	0.064	58.15	0	0.01	0	124.76	0	112.81	0	29.76	0	179.61	0	0.01
C142	-0.126	-214.24	-0.004	-0.01	-0.005	-11.13	-2.048	-48.01	0	-171.09	0	-200.1	-0.002	0
C142OH	0	-36.29	0	0	0	69.59	-1.172	-94.9	-0.058	-204.58	0	-76.89	-0.001	-0.01
C16	0.008	-13.43	0.002	0.01	-0.454	-159.11	0	111.71	0.014	115.85	0	188.15	0.004	0.01
C161	0.094	171.78	0.006	0.01	0.548	169.79	0	-35.09	0	-114.5	0	86.35	0	0.01
C161OH	0.009	2.73	0	0	0	33.54	0.84	184.43	0	130	0	43.96	0	0
C162	0	47.03	0	0.01	0	11.17	1.795	148.8	0	4.89	0	-25.6	0	0.01
C162OH	0	80.15	0	-0.01	0	-87.24	0	-184.01	-0.017	-64.99	0	-40.12	0	-0.01
C16OH	0.018	111.62	0.006	0.01	-0.182	-98.32	0	7.05	0	60.28	0	23.02	0	0.01
C18	-0.102	-186.58	-0.003	-0.01	-0.509	-247.86	0	-109.86	0.017	94.93	0	51.46	-0.002	-0.02
C181	0	27.05	0	0.02	0	40.09	0	-86.83	0.02	110.08	0	21.61	0	0.01
C181OH	0.04	127.52	0.008	0.01	0	-120.91	0.738	159.68	0	-100.55	0	-29.08	0	0.01
C182	-0.046	-213.79	-0.002	-0.01	0.389	212.66	0	-68.01	-0.113	-289.61	0	-164.11	-0.012	-0.02
LPC a C14:0	0.005	49.83	0	0	0.306	156.81	0	82.75	0.189	315.92	0.143	349.52	0.011	0.02
LPC a C16:1	0.042	131.85	0.009	0.02	0.376	261.27	0.308	19.1	0	-106.09	0	91.79	0.003	0.01
LPC a C17:0	0.133	301.31	0.026	0.05	0.407	212.69	3.257	319.97	0.193	529.75	0.312	627.88	0.072	0.08
LPC a C18:0	-0.192	-245.2	-0.034	-0.08	-0.358	-181.95	-2.78	-272.12	-0.092	-146.08	-0.383	-288.53	-0.073	-0.11
LPC a C18:2	-0.018	-66.42	-0.019	0	0	46.65	0	23.73	0	-104.07	0	-295.2	-0.001	-0.01
LPC a C20:4	-0.01	-82.39	0	-0.02	-0.114	-62.96	0	-23.98	0	6.55	0	-159.62	0	-0.02
LPC a C26:1	0	4.98	0	0.01	0.122	50.63	0	-19.13	0	-73.18	0	-116.46	-0.003	-0.01
LPC a C16:0	0	-152.47	0	0.03	0	14.74	0	-132.11	-0.11	-191.5	0	-133.41	0	0.02
LPC a C18:1	-0.098	-150.63	0	-0.04	0	-59.25	0	-6.83	0	2.42	0	-212.83	0	0
LPC a C20:3	0.184	288.57	0.02	0.05	0.167	174.48	0	20.04	-0.07	-221.4	0	52.74	0	0.04
LPC a C24:0	0	52.85	0	0	0.55	217.9	0	-50.3	-0.049	-149.67	-0.109	-208	0	0.01
LPC a C26:0	0	7.63	0	-0.02	0	-39.89	0	-96.27	-0.059	-218.71	0	5.24	-0.002	-0.02
LPC a C28:0	0	-16.18	0	0.01	-0.252	-204.87	0	32.4	0	-84.41	0	-32.69	-0.001	-0.01
LPC a C28:1	0	49.14	0	0.01	0.097	-14.28	0.056	133.17	0	39.57	0	108.73	0	0
PC aa C24:0	-0.037	-162.25	-0.009	-0.02	0	2.99	0	-107.47	0	147.45	0	-98.11	0	0.01
PC aa C26:0	0	-136.59	0	0	0	-80.87	0	-119.22	0	-128.99	0	-138.14	-0.009	-0.01
PC aa C28:1	0.237	503.43	0.033	0.05	0.715	432.29	2.086	495.2	0	244.06	0.182	684.64	0.037	0.05
PC aa C30:0	0	74.7	0	0	0.408	99.18	0	136.3	0	107.86	0	308.67	0	0.01
PC aa C32:0	0.038	66.49	0.007	0.03	0	-44.2	0	-35.79	-0.238	-405.31	0	31.77	0	0
PC aa C32:1	0	116.23	0	-0.02	0	-34.7	0.52	64.23	0	114.28	0.071	241.62	0.017	0.01
PC aa C32:2	0	50.38	0	-0.01	0	55.97	0	65.37	0	64.85	0	84.37	0	-0.01
PC aa C32:3	-0.06	-236.7	-0.011	-0.02	-0.058	-99.98	1.616	187.83	0	174.75	-0.04	-103.08	-0.005	-0.01
PC aa C34:1	0	-64.81	0	-0.08	-0.106	-42	0	-15.68	0	134.12	0	-4.39	0	-0.06
PC aa C34:2	0	-8.15	0	0.06	-0.133	-95.58	0	59.58	0	-51.91	0	-151.66	0	0.05
PC aa C34:3	0	39.81	0	0.02	0.062	108.11	0	43.26	0	-97.77	0	-73.79	0	0
PC aa C34:4	-0.077	-256.17	-0.011	-0.01	0	-2.87	0	-79.29	0	-86.08	0	-2.7	-0.009	-0.02
PC aa C36:0	0.035	95.41	0	0	0.411	293.32	-0.28	-24.18	0	-49.86	0	53.78	0	0.01
PC aa C36:1	-0.084	-168.33	0	0	-0.593	-273.28	-2.063	-190.8	0.05	126.25	0	-147.34	0	0.03
PC aa C36:2	0	-83.42	-0.002	-0.03	-0.507	-231.99	0	-113.02	0	23.37	-0.01	-314.38	0	-0.08

PC aa C36:3	0	-3.04	0	-0.1	0.327	150.78	0	12.89	0	-44.81	0	-16.27	0	-0.03
PC aa C36:4	-0.097	-205.5	0	0.03	0	36.5	0	100.49	0	-28.92	0	-55.05	0	0.01
PC aa C36:5	0	26.75	0	-0.01	0	-77.05	0	206.54	0	-49.92	0	-71.22	0	0.01
PC aa C36:6	0	-11.03	-0.001	-0.01	0	27.22	0	57.82	0	-87.77	0	39.91	0	-0.01
PC aa C38:0	0	24.39	0	0	0	24.71	-2.232	-275.55	0	3.7	0	-171.6	0	0
PC aa C38:1	-0.119	-318.28	-0.014	-0.02	0	32.44	0	-304.21	-0.044	-112.17	0	-165.86	-0.012	-0.02
PC aa C38:3	0.122	150.75	0.012	0.12	0	63.73	0	-119.52	0	-182.36	0	38.95	0	0.06
PC aa C38:4	0	29.09	0	-0.02	-0.202	-133.2	0	-36.61	0	1.11	0	-82.06	0	0
PC aa C38:5	0.013	101.73	0	0.01	0	-84.29	0	64.53	0	8.78	0	16.64	0	0.06
PC aa C38:6	-0.007	-106.98	0	0.04	0.01	119.78	0	-1.53	-0.012	-79.14	0	-73.68	0	0
PC aa C40:1	-0.011	-96.37	0	0	-0.294	-148.2	0	-11.9	-0.005	-10.19	0	-92.65	0	0
PC aa C40:2	0.028	37.18	0.003	0.01	0	48.83	0	75.22	-0.025	-136.21	0	-34.44	0	0
PC aa C40:3	-0.031	-88.19	-0.005	-0.01	0.24	123.85	0	208.16	-0.015	-32.9	-0.089	-309.67	0	0
PC aa C40:4	0.056	124.82	0.009	0.01	0	-56.75	0	104.13	0	-131.08	0	-53.62	0.009	0.01
PC aa C40:5	0.125	241.93	0.017	0.02	0.133	119.18	0	-192.57	0	154.82	0.051	216.52	0.011	0.02
PC aa C40:6	0	-64.23	0	0.02	0	-86.32	0	-270.37	-0.043	-75.47	0	-129.51	0	0
PC aa C42:0	0.05	199.52	0.007	0.01	0	-2.94	0	129.75	-0.056	-210.79	0	79.6	0	0
PC aa C42:1	0.022	188.37	-0.001	-0.01	0	-128.81	0	-4.48	0	-89.58	0	-175.1	-0.006	-0.01
PC aa C42:2	0	-6.74	0	0	-0.095	-66.04	0	-82.99	0	-1.31	0	42.78	0	0
PC aa C42:4	0.044	131.75	0.001	0.01	0	64.29	3.363	274.58	0	58.03	0	-145.03	0	0
PC aa C42:5	0	-29.75	0	0	-0.054	50.56	0.588	-49.46	-0.045	-94.45	0	-144.5	-0.003	-0.01
PC aa C42:6	0.012	159.58	0	0	0	-136.13	0.082	90.83	0	-74.67	0	182.96	0	0
PC ae C30:0	0	65.42	0	0	0.111	67.92	2.171	311.44	0.107	268.23	0.088	477.88	0.005	0.01
PC ae C30:1	-0.082	-205.51	0	0	-0.362	-224.57	0	-34.22	0	28.16	0	-168.72	0	0.01
PC ae C30:2	-0.09	-324.47	-0.009	-0.02	0	-60.33	0.265	17.86	0	224.69	0	76.93	-0.006	-0.01
PC ae C32:1	0	76.43	0	0.01	0	-59.25	0	42.16	0	-179.46	0	71.96	0	0.01
PC ae C32:2	0	17.13	0	-0.01	0	41.1	0	-29.93	0	92.8	0	-20.84	0	-0.01
PC ae C34:0	0	-51.38	0	-0.01	0	-161.65	1.029	193.32	0.147	235.9	0.087	443.69	0.002	0
PC ae C34:1	0.032	75.65	0.002	0.01	0	29.53	0	141.95	0.218	191.76	0	297.45	0.013	0
PC ae C34:2	0	84.87	0	0.01	0	16.96	0	43.72	0	137.1	0	112.92	0	0.01
PC ae C34:3	0	-148.93	-0.009	-0.02	-0.227	-96.51	-0.862	-326.25	0	-70.83	-0.047	-284.46	-0.012	-0.02
PC ae C36:0	-0.034	-116.25	0	0	0	1.66	-0.268	9.97	0.012	29.57	0	-34.52	0	0
PC ae C36:1	0.011	122.63	0	0	0	-25.56	0	-12.09	0.076	193.58	0	295.7	0.001	0
PC ae C36:2	0	96.48	0	0	0.242	95.98	2.873	379.4	0.073	323.72	0	394.67	0.008	0.02
PC ae C36:3	0	-130.43	0	0	-0.155	-112.49	0	-134.58	0	-92.28	0	-78.44	0	-0.01
PC ae C36:4	-0.029	-94.95	0	-0.03	-0.23	-152.3	-4.288	-300.52	0	108.91	0	-115.58	-0.01	-0.04
PC ae C36:5	0	-96.16	0	0.02	-0.29	-104.03	-0.459	-209.86	0	94.05	0	-96.56	0	0.02
PC ae C38:0	0	-128.58	-0.001	-0.01	0	17.3	0	88.27	-0.039	-129.92	-0.045	-258.8	-0.005	-0.02
PC ae C38:1	0	-42.56	0	0.09	0	46.62	0	27.75	0	86.11	0	13.48	0	0.07
PC ae C38:2	0	9.93	0	-0.07	0	-40.82	0.456	191.76	0	-72.58	0	-123.01	-0.009	-0.06
PC ae C38:3	0	53.42	0	0.01	0	-6.09	0	-137.04	0	155.78	0	255.9	0	0
PC ae C38:4	0.04	223.92	0.005	0.03	0	-5.96	0	183.27	0.064	292.49	0	291.57	0.013	0.04
PC ae C38:5	-0.111	-172.99	0	-0.01	-0.122	-96.41	-0.239	-181.11	0	20.86	0	-136.32	0	0
PC ae C38:6	0	35.02	0	0.01	0	-86.39	-0.195	-259.04	0	-97.57	0	-32.46	0	0
PC ae C40:1	0.025	175.29	0	0.01	0.343	14.33	0	-8.02	-0.048	-174.14	0	-127.14	0	0
PC ae C40:2	0	-122.84	-0.006	-0.01	-0.278	-219.35	-1.425	-139.46	0	94.14	0	-111.18	0	-0.04
PC ae C40:3	0	-45.62	0	0.05	-0.183	-168.17	0	-45.02	0	13.64	0	-260.18	0	0.1
PC ae C40:4	0	26.39	0	0.07	0.624	189.49	0	28.39	0	-61.58	0	-6.59	0	0.02
PC ae C40:5	-0.02	-141.58	-0.006	-0.04	0	25.6	0	170.4	0	-9.5	0	-39.11	0	-0.01
PC ae C40:6	0	-89.87	-0.001	-0.02	0.141	125.54	0	92.18	0	1.3	0	16.14	0	-0.01
PC ae C42:0	0.007	91.11	0	0	0.009	52.73	0	230.65	0	95.71	0	11.65	0	0
PC ae C42:1	0	62.13	0	-0.12	0.157	123.29	-0.689	-152.92	-0.074	-174.63	0	1.72	0	-0.07
PC ae C42:2	0.036	120.3	0	0.02	0	-23.41	0	109.64	-0.024	-0.83	0	15.27	0	-0.05
PC ae C42:3	0	22.88	0	0.02	-0.23	-155.17	0	-59.76	0	44.61	0	-110.44	0	0.01

PC ae C42:4	-0.039	-106.96	-0.001	-0.05	0.457	235.78	0	61.98	-0.034	-72.42	0	-87.55	-0.001	0
PC ae C42:5	0.069	214.6	0.003	0.01	-0.237	-93.56	0	134.27	0	-5.53	0	135.24	0	0
PC ae C44:3	-0.038	-200.43	-0.006	-0.01	-0.312	-139.43	-0.909	55.92	0	-96.16	0	-169.01	-0.008	-0.01
PC ae C44:5	0.021	3.2	0.005	0.01	-0.313	-207.98	0	-63.36	0	25.18	0	-152.54	0	0
PC ae C44:6	0.041	230.7	0.002	0.01	0.44	285.71	0	-124.62	-0.083	-189.55	0	-6.39	0	0
SM C16:0	-0.106	-157.73	-0.013	-0.02	-0.747	-268.59	0	-177.08	0	-205.66	-0.213	-303.77	-0.024	-0.03
SM C16:1	-0.291	-382.61	-0.055	-0.06	0	60.63	-0.841	-260.1	-0.124	-205.84	-0.486	-338.26	-0.053	-0.06
SM C18:0	0.052	236.86	0	0.01	0	-26.37	-0.845	-94.67	0	-103.76	0	31.36	0	0.01
SM C18:1	-0.08	-297.83	0	-0.01	0.076	130.97	-2.151	-275.4	0	-6.18	0	-142.57	0	0
SM C20:2	0.027	190.24	0.005	0.01	0	-28.96	0	89.18	0	57.59	0	-19.56	0	0
SM C24:0	-0.109	-341.7	-0.022	-0.02	-0.755	-303.44	-0.483	-63.07	-0.016	-119.42	-0.134	-452.16	-0.026	-0.03
SM C24:1	-0.018	-103.48	0	0	-0.354	-177.1	-0.982	-142.69	-0.072	-283.46	-0.241	-409.74	-0.008	-0.01
SM-OH C14:1	0.463	678	0.078	0.08	0.806	409.68	4.648	602.43	0.292	472.18	1.128	941.88	0.11	0.12
SM-OH C16:1	0.023	163.78	0	-0.01	0	82.31	1.156	312.93	0.157	365.19	0	480.35	0.004	0.01
SM-OH C22:1	-0.088	-245.53	-0.009	-0.02	0	-50.25	0	74.39	0	49.34	0	-51.79	0	-0.01
SM -OH C22:2	0.042	185.85	0.007	0.01	0	64.07	0	134.66	0	107.04	0	293.48	0.001	0.01
Hexose	0.051	200.26	0	0.01	-0.032	-76.05	0	168.22	0	-124.19	0	-97.98	0	0
age	-0.02	-4.63	0.001	0	0	33.65	-0.43	-105.29	-0.003	-12	-0.002	28.85	0.001	0
sex	-0.294	-153.42	0	-0.06	0.088	192.66	2.326	246.22	0	-1.68	0	9.28	0	0.05
test site	0.243	526.45	0.04	0.04	-0.027	-33.06	-0.024	-308.14	-0.272	-619.96	0	61.86	0	0
smoking status	-0.016	-137.92	0	-0.01	-1.678	-749.57	0	86.54	0.108	149.91	0	-97.31	0	0
physical activity	0.012	-16.9	0	0	0	-13.01	0	9	0	-5.74	0.009	-4.1	0	0
lipid-lowering medication	-0.011	-107.53	0	-0.03	-0.129	-85.38	0	30.8	0	-55.88	0	-33.29	0	-0.04
HRT	0	209.92	0	-0.04	-1.312	-609.86	-6.561	-378.87	0	86.27	0	44.11	-0.027	-0.02
BMI	0	-7.93	0.001	0	0	26.87	0.332	137.87	0	-59.71	0.05	114.37	0.003	0
intercept	0.062	5	0.738	0.96	-1.655	-50.37	0.284	27.67	-0.713	14.46	0.438	12.11	1.236	1.16

Supplemental Figure 4. Strength of associations of metabolites, socio-demographic and lifestyle covariates with consumption levels of total and types of dairy products in the derivation set of the Fenland study with the whole set of 174 metabolites. Colors show the relative strength of associations within each column. Intensity of red color indicates strength of positive associations and intensity of blue color indicates strength of negative associations. † Strengths of associations are expressed as the beta coefficients of elastic net penalized logistic regression and ordinary logistic regression. ‡ Strengths of associations are expressed as the beta coefficients of elastic net penalized linear regression and ordinary linear regression.

Fenland study

Metabolite	trans-OH-Pro	C0	C18	LPC a C14:0	LPC a C17:0	LPC a C18:0	LPC a C20:3	PC aa C32:0	PC ae C34:1	SM C16:0	SM-OH C14:1
trans-OH-Pro	1.00	0.28	0.13	0.06	0.04	0.11	0.17	0.06	0.04	0.06	-0.01
C0	0.28	1.00	0.29	0.22	0.15	0.27	0.32	0.12	0.09	0.13	0.08
C18	0.13	0.29	1.00	0.19	0.36	0.36	0.31	0.23	0.24	0.25	0.23
LPC a C14:0	0.06	0.22	0.19	1.00	0.41	0.40	0.51	0.36	0.38	0.13	0.25
LPC a C17:0	0.04	0.15	0.36	0.41	1.00	0.73	0.43	0.20	0.37	0.34	0.50
LPC a C18:0	0.11	0.27	0.36	0.40	0.73	1.00	0.59	0.24	0.20	0.39	0.30
LPC a C20:3	0.17	0.32	0.31	0.51	0.43	0.59	1.00	0.26	0.27	0.18	0.13
PC aa C32:0	0.06	0.12	0.23	0.36	0.20	0.24	0.26	1.00	0.70	0.53	0.42
PC ae C34:1	0.04	0.09	0.24	0.38	0.37	0.20	0.27	0.70	1.00	0.48	0.55
SM C16:0	0.06	0.13	0.25	0.13	0.34	0.39	0.18	0.53	0.48	1.00	0.74
SM-OH C14:1	-0.01	0.08	0.23	0.25	0.50	0.30	0.13	0.42	0.55	0.74	1.00

EPIC-Norfolk study

Metabolite	trans-OH-Pro	C0	C18	LPC a C14:0	LPC a C17:0	LPC a C18:0	LPC a C20:3	PC aa C32:0	PC ae C34:1	SM C16:0	SM-OH C14:1
trans-OH-Pro	1.00	-0.06	0.16	0.03	-0.14	-0.03	0.10	0.08	0.06	0.07	0.00
C0	-0.06	1.00	0.05	0.37	0.09	0.61	0.25	0.27	0.03	-0.04	0.25
C18	0.16	0.05	1.00	0.07	0.00	0.05	0.09	0.04	0.08	0.04	0.03
LPC a C14:0	0.03	0.37	0.07	1.00	-0.07	0.57	0.41	0.47	-0.05	0.09	0.54
LPC a C17:0	-0.14	0.09	0.00	-0.07	1.00	0.10	0.06	0.11	0.35	-0.20	0.12
LPC a C18:0	-0.03	0.61	0.05	0.57	0.10	1.00	0.42	0.32	-0.04	-0.01	0.60
LPC a C20:3	0.10	0.25	0.09	0.41	0.06	0.42	1.00	0.16	0.23	0.09	0.43
PC aa C32:0	0.08	0.27	0.04	0.47	0.11	0.32	0.16	1.00	-0.13	0.09	0.55
PC ae C34:1	0.06	0.03	0.08	-0.05	0.35	-0.04	0.23	-0.13	1.00	-0.12	-0.19
SM C16:0	0.07	-0.04	0.04	0.09	-0.20	-0.01	0.09	0.09	-0.12	1.00	0.10
SM-OH C14:1	0.00	0.25	0.03	0.54	0.12	0.60	0.43	0.55	-0.19	0.10	1.00

Supplemental Figure 5. Correlation matrices of the selected top metabolites associated with at least one of each of milk, yogurt, cheese, butter or total dairy consumption in the Fenland and EPIC Norfolk studies. Selection of top metabolites was based on the criterion of absolute values of elastic net coefficients >mean+ 2×SD of all the 82 coefficients. EPIC: European Investigation into Cancer and Nutrition
